# Supplementary material for: Aetiology and severity of childhood pneumonia in primary care in Malawi: a cohort study
Source: BMJ Open. 2021 Jul 29;11(7):e046633. doi: 10.1136/bmjopen-2020-046633 (PMC8323352; doi:10.1136/bmjopen-2020-046633)
Supplement: Supplementary data [file bmjopen-2020-046633supp001.pdf]

Supplemental file

Aetiology and Severity of Childhood Pneumonia in Primary Care in Malawi: A Cohort Study

Table of contents

S1: Study Location and Background health data **3**

S2: Microbiology **4**
S2a Microbiology techniques 4
Table S1 Characteristics of individual children with bacterial pneumonia 5
Table S2 Microbiology Results By month 6
Table S3 Microbiology Results By age 7

S3: Markers of severity **8**
Figure S1 Difficulty DRAWING score 8

S4: List of BIOTOPE team & Primary Affiliation **8**

S1: Study Location and Background health data

Mzuzu is one of the four cities in Malawi, with a population of 221,272 (National Statistics Office, 2018). Mzuzu health centre also known as Mapale or Mzimba North Health centre is stituated at the City centre of Mzuzu city while Mzuzu Central Hospital is situated at around 4 km Northwards of the city. The health centre currently serves almost the whole city as a primary health care point while the central hospital is regional referral tertiary hospital serving six districts in the northern region, over hundred health centres and serving a population of over 1.9 million people. It also serves as a secondary healthcare facility for the local (Mzuzu city) population. The majority (98.0%) of our participants were from Mapale health centre. The Central hospital initially served as a primary healthcare facility also but prior to commencement of the study the local health authority decided to maintain it as a secondary care facility only. Therefore it introduced a "bypass fee" where patients who attended the hospital clinics without being referred from a health centre/district hospital were charged MK 1,500 (approxmately 2 Euros). This led to Mapale becoming the main site of recruitment due to the focus on recruiting patients from primary care and before they had received any treatment.

UNICEF data from 2013 reports an under five mortality rate of 68 per 1,000 live births and under one mortality rate of 44 per 1,000 live births in Malawi. In 2016 the estimated coverage was 83% for pneumococcal conjugate vaccine and 84% for Haemophilus influenza type B vaccine in Malawi,[11]

HIV prevalence in antenatal clinics in the Mzimba district (where Mzuzu is located) among those who accepted testing was 9.1%[1]. A study in 2011 in Malawi showed that of 5,068 samples from infants <3 months of age , 764 were ELISA positive indicating 15.1% (14.1–16.1%) of mothers were HIV-infected and passed antibodies to their infant. Sixty-five of the ELISA-positive samples tested positive by DNA PCR, indicating a vertical transmission rate of

8.5% (6.6–10.7%). Survey data indicates 64.8% of HIV-infected mothers and 46.9% of HIV-exposed infants received some form of antiretroviral prophylaxis[2].

S2: Microbiology

S2a Microbiology techniques

Blood cultures

The blood culture samples were inoculated into the Bactec Peds Plus/F Culture vial (Becton Dickinson Diagnostic Instrument Systems). These bottles were incubated in an automated blood culture Bactec™ 9050 which was set at protocol limits of maximum of 7 days to declare the sample negative if no growth has been detected. All positive blood cultures were further subcultured on to agar plates and identified using traditional phenotypic methods (Blood agar, MacConkey and Chocolate agar plate) and Vitek 2 microbial detection system (Biomerieux)

Bacterial PCR

The Masterpure™ complete DNA and RNA purification kit as well as QIA Symphony Automated DNA extraction system [manufacturer. Country] which is an automated method was used to extract DNA and RNA. The extracted DNA was run on AB7500 instruments as per previously detailed methods [4,5]

Viral RT-PCR

Viral RT-PCR was performed on nasopharyngeal and oropharyngeal swabs using a Luminex® MAGPIX® instrument [manufacturer, country] with xPONENT® and SYNCT™ software ,in line with manufacturer's instructions. The panel used was NxTAG respiratory pathogen. The

pathogens targeted in this assay were; Influenza A, Influenza A H1, Influenza B, Influenza A H3, Respiratory Syncytial Virus A, Respiratory Syncytial Virus B, Coronavirus 229E, Coronavirus OC43, Coronavirus NL63, Coronavirus HKU1, Human Metapneumovirus, Rhinovirus/Enterovirus Adenovirus, Parainfluenza 1, Parainfluenza 2, Parainfluenza 3, Parainfluenza 4, Human Bocavirus, *Chlamydophila pneumoniae, Mycoplasma pneumoniae, Legionella pneumophila*

Table S1 Characteristics of individual children with bacterial pneumonia

Bacteria	Method of detection	Age (years)	Gender	Malaria	HIV	Antibiotic
S. pneumoniae & Haemophilus spp.	Blood PCR	1.1	M	Neg	Neg	Cotrimoxazole
S. pneumoniae	Blood PCR	2.3	M	Pos	Neg	Cotrimoxazole
S. pneumoniae & Haemophilus spp.	Blood PCR	1.1	M	Pos	Neg	Cotrimoxazole
S. pneumoniae & Haemophilus spp.	Blood PCR	1.7	M	Neg	Neg	Unknown
S aureus	Blood culture	2.4	M	Neg	Neg	Cotrimoxazole
S aureus	Blood culture	1.7	M	Neg	Neg	Cotrimoxazole
Haemophilus spp.	Blood PCR	2.8	F	Neg	Neg	Unknown
Haemophilus spp.	Blood PCR	2.7	M	Neg	Neg	Amoxicillin
C. pneumoniae	OP/NP swab PCR	3.6	M	Neg	Neg	Amoxicillin
M. pneumoniae	OP/NP swab PCR	2.6	F	Neg	Pos	Amoxicillin
M. pneumoniae	OP/NP swab PCR	1.7	M	Neg	Neg	Amoxicillin

M. pneumoniae	OP/NP swab PCR	2.4	F	Neg	Neg	Unknown
C. pneumoniae	OP/NP swab PCR	0.6	M	Neg	Unk	Amoxicillin

Table S2 Microbiology Results By month

Variable	Statistic	Mar	Apr	May	Jun
**Bacterial**					
S. pneumoniae	n (%)	0/12 (0%)	0/40 (0%)	3/185 (1.6%)	1/257 (0.4%)
S. auerus	n (%)	0/12 (0%)	0/40 (0%)	0/185 (0%)	2/257 (0.4%)
Haemophilus spp.	n (%)	0/12 (0%)	0/40 (0%)	2/185 (1.1%)	3/257 (1.2%)
M. pneumoniae	n (%)	0/12 (0%)	1/40 (2.5%)	2/185 (1.1%)	0/257 (0%)
C._pneumoniae	n (%)	0/12 (0%)	0/40 (0%)	1/185 (0.5%)	1/257 (0.4%)
Other_culture	n (%)	0/12 (0%)	0/40 (0%)	1/185 (0.5%)	2/257 (0.8%)
**Viral[1]**					
Adenovirus	n (%)	6/12 (50%)	10/40 (25%)	33/185 (17.8%)	30/257 (11.7%)
Bocavirus	n (%)	2/12 (16.7%)	7/40 (17.5%)	37/185 (20%)	47/257 (18.3%)
CoV* HKU	n (%)	0/12 (0%)	0/40 (0%)	0/185 (0%)	0/257 (0%)
CoV* NL63	n (%)	0/12 (0%)	1/40 (2.5%)	0/185 (0%)	0/257 (0%)
CoV* 229E	n (%)	0/12 (0%)	0/40 (0%)	0/185 (0%)	1/257 (0.4%)
CoV* OC43	n (%)	0/12 (0%)	0/40 (0%)	0/185 (0%)	1/257 (0.4%)
Influenza A	n (%)	0/12 (0%)	10/40 (25%)	42/185 (22.7%)	23/257 (8.9%)
Influenza AH1	n (%)	0/12 (0%)	10/40 (25%)	37/185 (20%)	16/253 (6.2%)
Influenza AH3	n (%)	0/12 (0%)	0/40 (0%)	3/185 (1.6%)	3/257 (1.2%)
Influenza B	n (%)	0/12 (0%)	0/40 (0%)	2/185 (1%)	6/257 (2.3%)
HMPV[+]	n (%)	2/12 (16.7%)	10/40 (25%)	33/185 (17.8%)	27/257 (10.5%)

Parainfluenza 1	n (%)	0/12 (0%)	2/40 (5%)	5/182 (2.7%)	4/257 (1.5%)
Parainfluenza 2	n (%)	0/12 (0%)	3/40 (7.5%)	4/185 (2.2%)	3/257 (1.2%)
Parainfluenza 3	n (%)	1/12 (8.3%)	0/40 (0%)	3/185 (1.6%)	0/257 (0%)
Parainfluenza 4	n (%)	1/12 (8.3%)	3/40 (7.5%)	7/185 (3.8%)	5/257 (1.9%)
RSV// A	n (%)	0/12 (0%)	1/40 (2.5%)	22/185 (11.9%)	129/257 (50.2%)
RSV// B	n (%)	0/12 (0%)	2/40 (5%)	12/185 (6.5%)	35/257 (13.6%)
Rhino/enterovirus	n (%)	10/12 (83.3%)	22/40 (55%)	132/185 (71.3%)	128/257 (49.8%)

* = Coronavirus

+ = Human metapneumovirus

// = Respiratory syncytial virus

Table S3 Microbiology Results By age

Variable	Statistic	2-11 months	12-35 months	36+ months
**Bacterial**				
S. pneumoniae	n (%)	2/157 (1.3%)	2/252 (0.8%)	0/85 (0%)
S. auerus	n (%)	0/157 (0%)	2/252 (0.8%)	0/85 (0%)
Haemophilus spp.	n (%)	2/157 (1.4%)	3/252 (1.2%)	0/85 (0%)
M. pneumoniae	n (%)	0/157 (0%)	3/252 (1.2%)	0/85 (0%)
C. pneumoniae	n (%)	0/157 (0%)	1/252 (0.4%)	1/85 (1.2%)
**Viral**				
Adenovirus	n (%)	12/157 (7.6%)	48/252 (19%)	17/85 (20%)
Bocavirus	n (%)	25/157 (15.9%)	52/252 (20.6%)	14/85 (16.5%)
CoV* HKU	n (%)	0/157 (0%)	0/252 (0%)	0/85 (0%)
CoV* NL63	n (%)	0/157 (0%)	1/252 (0.4%)	0/85 (0%)
CoV* 229E	n (%)	0/157 (0%)	1/252 (0.4%)	0/85 (0%)
CoV* OC43	n (%)	0/157 (0%)	1/252 (0.4%)	0/85 (0%)
Influenza A	n (%)	8/157 (5%)	40/252 (15.9%)	26/85 (30.6%)
Influenza AH1	n (%)	8/157 (5%)	30/252 (11.9%)	24/85 (28.2%)
Influenza AH3	n (%)	0/157 (0%)	5/252 (1.9%)	1/85 (1.2%)
Influenza B	n (%)	2/157 (1.3%)	3/252 (1.2%)	2/84 (2.3%)
HMPV+	n (%)	19/157 (12.1%)	41/252 (16.3%)	11/84 (12.9%)
Parainfluenza 1	n (%)	2/157 (1.3%)	4/252 (1.6%)	5/85 (5.8%)
Parainfluenza 2	n (%)	2/157 (1.3%)	5/252 (2%)	2/85 (2.3%)
Parainfluenza 3	n (%)	1/157 (0.6%)	3/252 (1.2%)	0/85 (0%)

Parainfluenza 4	n (%)	4/157 (2.5%)	9/252 (3.6%)	3/85 (3.5%)
RSV// A	n (%)	61/157 (38.8%)	76/252 (30.2%)	10/85 (11.8%)
RSV// B	n (%)	18/157 (11.4%)	22/252 (8.7%)	7/85 (8.2%)
Rhino/enterovirus	n (%)	83/157 (52.9%)	148/252 (58.7%)	53/85 (62.3%)

* = Coronavirus

+ = Human metapneumovirus

// = Respiratory syncytial virus

S3: Markers of severity

Figure S1 Difficulty DRAWING Breath score and risk of hospitalisation

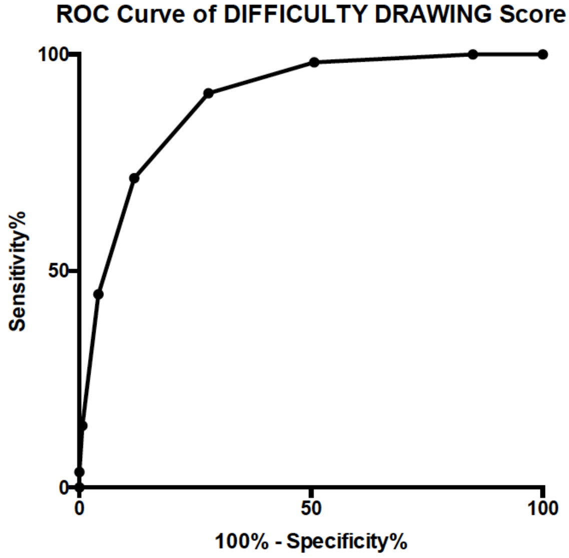

DIFFICULTY DRAWING breath score = 1 for each of:

DIFFICULTY breathing
Deep breathing
Respiratory Rate > 70 bpm

Age <2 years
Wheeze
INdrawing lower chest wall
Grunting

Area under the ROC curve
Area 0.8918
Std. Error 0.02002
95% confidence interval 0.8525 to 0.9310
P value< 0.0001
Data
Controls (Not Hospitalised) 438
Patients (Hospitalised) 56
Missing Controls0
Missing Patients0

[1] The DD Score (strongly, positively associated) and previous number of vaccines (modestly, negatively associated) are the only independent predictors of hospitalisation in the model with the following covariates included:

- Staff designation of severe pneumonia (IMCI criteria)
- Age
- HIV status (immunosuppression)
- Completed WHO vaccination schedule
- Number sleeping in same room as child (overcrowding)
- Chimney present for indoor fire (indoor smoke)
- Electricity present (marker of socioeconomic status)
- Malaria co –infection (disease severity)
- Distance to nearest clinic (marker of healthcare access)

For each unit increase in the DD Score there is a 10.6% (95% CI 7.9-13.4) increase in the likelihood of severe disease as defined by hospitalisation (p<0.0001)

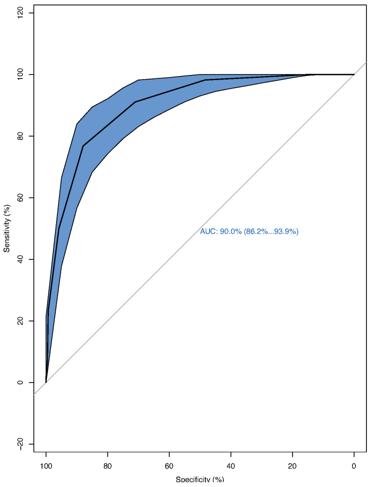

[2] The DD Score is the only strong, independent predictor of severe pneumonia as designated by the staff in a similar model with the following covariates included:

- Hospitalisation
- Age
- HIV status (immunosuppression)
- Completed WHO vaccination schedule
- Number sleeping in same room as child (overcrowding)
- Chimney present for indoor fire (indoor smoke)
- Electricity present (marker of socioeconomic status)
- Malaria co –infection (disease severity)
- Distance to nearest clinic (marker of healthcare access)

For each unit increase in the DD Score there is a 6.0% (95% CI 2.5-9.6) increase in the likelihood of severe disease as defined by WHO criteria (p<0.001)

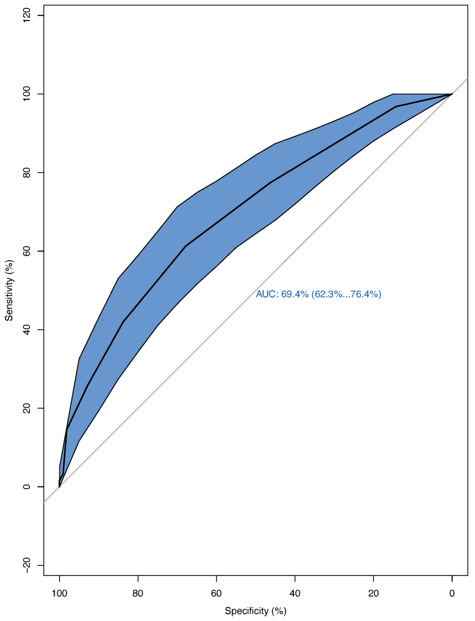

Area under the ROC curve
Area 0.69
95% confidence interval 0.62 to 0.76
P value< 0.001

S4: List of BIOTOPE team & Primary Affiliation

gHealth Research Group, School of Medicine, UCD
Joe Gallagher
Nadezhda Glezeva
Mark Ledwidge (statistics)

John O Donoghue
Dermot Wildes

Irish Meningitis and Sepsis Reference Laboratory
Richard Drew
Desiree Bennett
Nicola O'Sullivan

Luke International
Joseph Wu
Hsin yi Lee

Mzuzu Central Hospital/Mapale Health Centre
Master Chisale
Maynard Khondowe,
Themba Luwe
Hastings Scott
Chikondi Chimbatata
Cecilia Mwalilino
Kelvin Ulili
Lameck Manda
Joseph Phiri
Alinafe Mpicha
Isaac Namwiri

National Virus Reference Laboratory, Ireland
Cillian De Gascun
Suzie Coughlan
Linda Dunford
Christina Byrne

Queens University Belfast
Chris Watson

Royal College of Surgeons in Ireland
Sudip Das

Eoin O Connell (statistics)

1. Manda S, Masenyetse L, Cai B, Meyer R. Mapping HIV prevalence using population and antenatal sentinel-based HIV surveys: a multi-stage approach. Popul Health Metr 2015;13:22.
2. Sinunu MA, Schouten EJ, Wadonda-Kabondo N, et al. Evaluating the impact of prevention of mother-to-child transmission of HIV in Malawi through immunization clinic-based surveillance. PloS one 2014;9:e100741.
3. International. NSONMaI. Malawi Demographic and Health Survey 2015-16: Key Indicators Report. 2016.
4. Meyler KL, Meehan M, Bennett D, Cunney R, Cafferkey M. Development of a diagnostic real-time polymerase chain reaction assay for the detection of invasive Haemophilus influenzae in clinical samples. Diagn Microbiol Infect Dis. 2012 Dec;74(4):356-62. doi: 10.1016/j.diagmicrobio.2012.08.018. Epub 2012 Sep 25. PMID: 23017260.
5. [1]
Murphy J, O' Rourke S, Corcoran M, O' Sullivan N, Cunney R, Drew R. Evaluation of the Clinical Utility of a Real-time PCR Assay for the Diagnosis of Streptococcus pneumoniae Bacteremia in Children: A Retrospective Diagnostic Accuracy Study. Pediatr Infect Dis J. 2018 Feb;37(2):153-156. doi: 10.1097/INF.0000000000001772. PMID: 29076932.
