# Supplementary material for: Aetiology and severity of childhood pneumonia in primary care in Malawi: a cohort study
Source: BMJ Open. 2021 Jul 29;11(7):e046633. doi: 10.1136/bmjopen-2020-046633 (PMC8323352; doi:10.1136/bmjopen-2020-046633)
Supplement: Author's manuscript [file bmjopen-2020-046633.draft_revisions.pdf]

BMJ Open is committed to open peer review. As part of this commitment we make the peer review history of every article we publish publicly available.

When an article is published we post the peer reviewers' comments and the authors' responses online. We also post the versions of the paper that were used during peer review. These are the versions that the peer review comments apply to.

The versions of the paper that follow are the versions that were submitted during the peer review process. They are not the versions of record or the final published versions. They should not be cited or distributed as the published version of this manuscript.

BMJ Open is an open access journal and the full, final, typeset and author-corrected version of record of the manuscript is available on our site with no access controls, subscription charges or pay-per-view fees (http://bmjopen.bmj.com).

If you have any questions on BMJ Open's open peer review process please email info.bmjopen@bmj.com

BMJ Open

Aetiology and Severity of Childhood Pneumonia in Primary Care in Malawi

Journal:	*BMJ Open*
Manuscript ID	bmjopen-2020-046633
Article Type:	Original research
Date Submitted by the Author:	05-Nov-2020
Complete List of Authors:	Gallagher, Joe; University College Dublin College of Health Sciences, gHealth Research Institute<br>Chisale, Master; Mzuzu Central Hospital<br>Das, Sudipto; Royal College of Surgeons in Ireland<br>Drew, Richard; Irish Meningitis and Sepsis Reference Laboratory<br>Gleseva, Nadezhda; University College Dublin College of Health Sciences, gHealth Research Institute<br>Wildes, Dermot; University College Dublin College of Health Sciences, gHealth Research Institute<br>De Gascun, Cillian; National Virus Reference Laboratory<br>Wu, Tsung-Shu; Luke International Norway; Overseas Mission Department, Pingtung Christian Hospital<br>Ledwidge, Mark; University College Dublin College of Health Sciences, gHealth Research Institute<br>Watson, Chris; Queen's University Belfast Wellcome-Wolfson Institute for Experimental Medicine
Keywords:	Epidemiology < TROPICAL MEDICINE, Microbiology < PATHOLOGY, Paediatric infectious disease & immunisation < PAEDIATRICS, Paediatric thoracic medicine < PAEDIATRICS, PRIMARY CARE

SCHOLARONE™
Manuscripts

BMJ

I, the Submitting Author has the right to grant and does grant on behalf of all authors of the Work (as defined in the below author licence), an exclusive licence and/or a non-exclusive licence for contributions from authors who are: i) UK Crown employees; ii) where BMJ has agreed a CC-BY licence shall apply, and/or iii) in accordance with the terms applicable for US Federal Government officers or employees acting as part of their official duties; on a worldwide, perpetual, irrevocable, royalty-free basis to BMJ Publishing Group Ltd ("BMJ") its licensees and where the relevant Journal is co-owned by BMJ to the co-owners of the Journal, to publish the Work in this journal and any other BMJ products and to exploit all rights, as set out in our licence.

The Submitting Author accepts and understands that any supply made under these terms is made by BMJ to the Submitting Author unless you are acting as an employee on behalf of your employer or a postgraduate student of an affiliated institution which is paying any applicable article publishing charge ("APC") for Open Access articles. Where the Submitting Author wishes to make the Work available on an Open Access basis (and intends to pay the relevant APC), the terms of reuse of such Open Access shall be governed by a Creative Commons licence – details of these licences and which Creative Commons licence will apply to this Work are set out in our licence referred to above.

Other than as permitted in any relevant BMJ Author's Self Archiving Policies, I confirm this Work has not been accepted for publication elsewhere, is not being considered for publication elsewhere and does not duplicate material already published. I confirm all authors consent to publication of this Work and authorise the granting of this licence.

For peer review only

Aetiology and Severity of Childhood Pneumonia in Primary Care in Malawi

Joe Gallagher, MD[1*], Master Chisale, MSc[1,2], Sudipto Das, PhD[3], Richard Drew, MD[3,4], Nadezhda Glezeva, PhD[1], Dermot M Wildes[1], MB, Cillian De Gascun, MD[5], Tsung-Shu Joseph Wu, MSc[6,7], Mark Ledwidge, PhD[1], Chris J Watson, PhD[1,8].

On behalf of the BIOTOPE team

[1]gHealth Research Group, UCD Conway Institute, School of Medicine, University College Dublin, Ireland.

[2]Mzuzu Central Hospital, Mzuzu, Malawi.

[3]Royal College of Surgeons in Ireland, Dublin, Ireland.

[4] Irish Meningitis and Sepsis Reference Laboratory, Temple Street, Dublin 2.

[5] UCD National Virus Reference Laboratory, University College Dublin, Ireland.

[6] Luke International Norway, Mzuzu, Malawi.

[7]Overseas Mission Department, Pingtung Christian Hospital, Taiwan.

[8] Wellcome-Wolfson Institute for Experimental Medicine, Queen's University Belfast, Northern Ireland.

For peer review only - http://bmjopen.bmj.com/site/about/guidelines.xhtml

* Corresponding author:

Dr. Joe Gallagher,

School of Medicine,

University College Dublin,

Dublin.

Tel: 053 9421446

Fax: 053 9422526

Email: joejgallagher@gmail.com

For peer review only

Manuscript word count: 2,358 (Excluding Abstract, Tables, Figures & References)

Ethics - The protocol and related documents were successfully vetted by the National Health Science Research Committee of Malawi - Ethics approval number 15/11/1532

For peer review only - http://bmjopen.bmj.com/site/about/guidelines.xhtml

Abstract

Objective:

To determine the aetiology of community acquired pneumonia in children presenting to primary care in Northern Malawi, and to ascertain predictors for identification of children requiring hospitalisation.

Design: The BIOmarkers TO diagnose PnEumonia (BIOTOPE) study was a prospective cohort study conducted from March to June 2016.

Setting: Primary care in Northern Malawi.

Patients: 494 children aged 2 months to 59 months with World Health Organization defined pneumonia.

Main Outcome(s) and Measure(s): Number of children with bacterial infection identified and the sensitivity/specificity of WHO markers of severity for need for hospitalisation.

Results:

13 (2.6%) children had a bacterium consistent with pneumonia identified. A virus consistent with pneumonia was identified in in 448 (90.7%) of children. 56 children were admitted to hospital and two children died within 30 days. 442 (89.5%) received antibiotic therapy. Eleven children (2.6%) had HIV. WHO severity markers at baseline demonstrated poor sensitivity for the need for hospitalization with a sensitivity of 0.303 (95% confidence interval 0.188-0.441) and a specificity 0.9 (95% confidence

For peer review only - http://bmjopen.bmj.com/site/about/guidelines.xhtml

interval 0.868 to 0.926). A prediction rule to indicate the need for hospitalization was developed.

Conclusions and Relevance:

The low rate of bacterial infection and high use of antibiotics in the setting of high immunisation rates highlights the changing profile of childhood pneumonia. Similarly, the markers of need for hospitalization may have changed in the setting of extended immunisation. Further studies are required to examine this.

Article Summary:

- The pneumococcal vaccine was introduced in 2012 in Malawi and this study provides data on the aetiology and severity markers in primary care following the introduction of this vaccine.
- It demonstrates the significant reduction in bacterial infection compared to the previous studies undertaken using similar methods prior to introduction of this vaccine in Africa
- It also highlights that the severity markers in childhood pneumonia are changing and this is important since these markers are still used in primary care in Africa for the determination of referral to hospital.
- The study was in an urban area with high immunisation rates over one season and the lack of a control group (i.e., well children) limited our ability to verify the clinical significance of most viral pathogens and atypical bacteria

For peer review only - http://bmjopen.bmj.com/site/about/guidelines.xhtml

INTRODUCTION

Despite recent advances in reducing childhood mortality, pneumonia remains a major cause of morbidity and mortality among children aged under five years, with an estimated 935,000 deaths per annum[1]. Allied to this is the growing concern regarding antimicrobial resistance driven by overuse of antibiotics[2,3] and intercontinental spread of this resistance[4]. The increased use of universal immunisation, including haemophilus influenzae type B (Hib) and pneumococcal vaccines are likely to have altered the proportion with viral illness. This may also have altered the aetiology, severity, and host response in childhood pneumonia. A greater understanding and characterisation of these factors in primary care is required to continue advances in reducing childhood mortality from pneumonia.

There is limited information on non-malarial febrile illness in Africa[5]. However, where investigation for a bacterial diagnosis has been undertaken, few patients have documented bacterial infections[6-8]. Current strategies for the management of childhood pneumonia in developing countries focus on the identification of the illness and provision of antibiotics, despite the low reported prevalence of bacterial infection. Recent research also suggests that the rise in malaria rapid diagnostic testing may be leading to increased antibiotic use[9].

For peer review only - http://bmjopen.bmj.com/site/about/guidelines.xhtml

Most studies have focused on the hospitalised population, and yet in developing countries, it is in the community where these patients are most likely to present. Therefore, contemporary estimates of the microbiological causes of pneumonia and predictors of hospitalization would be of benefit. The use of microbiological techniques is hampered by the high rate of carriage of bacteria and viruses in children[10]. Finally, WHO markers of severity, which are used to guide referral for hospitalization in primary care, are currently based on clinical symptoms alone but the wider availability of technology allows the use of markers such as pulse oximetry to help determine severity.

The BIOTOPE study (BIOmarkers TO diagnose PnEumonia) was a prospective cohort study which sought to determine the aetiology of community acquired pneumonia in children presenting in primary care in Northern Malawi and identify predictors of children requiring hospitalization.

METHODS

Study Sites & Population

From March to June 2016, we recruited children at Mapale Health Centre and the outpatient department of Mzuzu Central Hospital in Mzuzu, Northern Malawi – both of which serve as primary care facilities for this urban area. Shortly after study commencement, the outpatient department of Mzuzu Central Hospital ceased to

For peer review only

provide primary care services. As such, 98% of participants were recruited from Mapale Health Centre.

Study participants:

Children presenting to the primary care facilities were assessed for study enrolment. Children aged 2-59 months were deemed eligible to participate in the study if the main presenting complaint was cough or difficulty breathing associated with tachypnoea (defined as >50 breaths per minute if aged 2-11 months or > 40 breaths per minute age if aged 12-59 months) or chest in-drawing. This is the current WHO clinical case definition of pneumonia[11]. Exclusion criteria listed were as follows: those discharged from hospital in the preceding 30 days; those who had completed a course of antibiotics within 14 days of presentation; or those who had received antibiotics prior to clinical assessment for this illness. The study details were discussed with a parent/guardian of any patient who fit our inclusion criteria, who in turn provided informed consent in a written format. The protocol and related documents were approved by the National Health Science Research Committee of Malawi and the Mzuzu Central Hospital Research Committee. Patients received timely treatment and supportive care as required. The study was conducted in compliance with the ethical standards as outlined by two ethics committees and was aligned with the values outlined by the Declaration of Helsinki. (National Health Research Committee of Malawi Ethics Approval #15/11/1532.

Study Procedures

For peer review only - http://bmjopen.bmj.com/site/about/guidelines.xhtml

Clinical Assessment

In order to record clinical data, a standardized report form was provided to our investigators incorporating demographics, risk factors, and items relevant to a pneumonia presentation. Immunisation records are held by patients in a patient held record known as the “health passport”. Only those who had a health passport with recorded immunisations were used for purpose of recording immunisation status. Where possible, children were followed up by contacting a caregiver at 7 days and 30 days following initial assessment by telephone. All children had a full blood count and malaria rapid diagnostic test. HIV rapid diagnostic testing was offered to all children aged over 12 months and HIV PCR testing to all children aged 2 months to 11 months.

Microbiologic and Other Investigations

For all patients, blood samples (8 ml) and pooled naso/oro-pharyngeal swabs were collected for microbiological investigation on site, and for further serologic and molecular analysis in Ireland. A systematic set of investigations were performed for all patients. Blood cultures were processed locally. Blood samples for PCR for *Streptococcus pnuemoniae*, *Staphylococcus aureus* and *Haemophilus influenzae* along with naso/oro-pharyngeal swabs for respiratory viruses and atypical bacteria were processed in Ireland.

Definitive Diagnosis Based on Predefined Criteria

The final diagnosis (in some cases, diagnoses) was ascertained for each patient using pre-determined criteria for both clinical and microbiological assessment. A case was

For peer review only - http://bmjopen.bmj.com/site/about/guidelines.xhtml

deemed to be “severe” should the child's condition fulfil WHO criteria for this state. Bacterial pneumonia was diagnosed on a positive blood culture or positive blood PCR for a relevant organism or positive nasopharyngeal swab for C. pneumoniae or M. pneumonia. Laboratory data were combined with the predetermined clinical criteria to establish each diagnosis.

Statistical Analyses

Data were entered into REDCap electronic data capture tools[14]. All statistical analyses were performed using R software (R Foundation for Statistical Computing). Sensitivity and specificity of WHO severity markers for hospitalization were calculated, with the Clopper-Pearson method used for their confidence intervals. Logistic regression using pre-defined variables was undertaken to identify a new clinical prediction model and the AUC was calculated by area summation, Tjur r-squared estimate and Hanley & McNeil's (1982) SE formula was used for AUC confidence intervals. Bootstrap analysis was performed using 1,000 iterations with means and 95% confidence intervals reported across the replications. A simplified clinical prediction rule was developed by selecting independent predictors of hospitalization measurable from multivariable analyses, allocating a single point for each characteristic and creating a sum score for each patient. We applied Receiver Operated Characteristic (ROC) analysis at various point thresholds of the score.

Patient & Public Involvement Statement

Patients were not involved in concept design, participant recruitment or study conduct at any stage from inception to completion of this project.

For peer review only - http://bmjopen.bmj.com/site/about/guidelines.xhtml

RESULTS

Study Sample

A total of 615 children were approached for inclusion in this study. (Figure 1). Demographic and clinical characteristics of the children included in the study are described in Table 1. At day seven, 225 caregivers were contactable by phone and 100% of children were alive. At day thirty, 195 caregivers were contactable and two children had died.

Immunisations

317 patients had a patient passport with immunisation record available. 239 of these patients were over the age of 12 months, and 109 were over 24 months of age. 98 (89.9%) of those aged 24 months or over who had immunisation records available had received full immunisations.

Microbiologic Analyses

A bacterium consistent with pneumonia was identified in 13 (2.6%) children. In three cases, more than one bacterial cause was identified in the same child. Respiratory viruses were found in 12 of the 13 children considered to have bacterial pneumonia. Viral causes alone were identified in 448 (90.7%) of children. Full details of the microbiological analyses are provided in Table 2. In 33 (6.7%) cases, no organism associated with pneumonia was identified.

For peer review only - http://bmjopen.bmj.com/site/about/guidelines.xhtml

WHO Severity Criteria and Hospitalization

WHO severity signs and other variables were recorded prospectively. The sensitivity and specificity of these are in Table 3.

Using logistic regression, further models were developed with significant improvement in discriminant value. In multivariable modeling, the following seven variables were predictive of hospitalization: Difficulty breathing, Deep breathing, Respiratory rate, Age, Wheeze, Lower chest wall indrawing. The AUC of the fully adjusted model for prediction of hospitalization was equal to 0.92 (95% CI 0.90, 0.93, p<0.0001). To create a simplified version of this model, we assigned a score of 1 to each sign or symptom (including categorical treatment of Age <2 years and Respiratory Rate >70 bpm) and generated a simple, 7 point "DIFFICULTY DRAWING breath" score for each patient, which predicted need for hospitalization with an AUC of 0.91 (95% CI 0.87 to 0.95, p<0.0001). A score of 0-1 was associated with low need for hospitalization, 2-3 was intermediate and a score of 4 or greater suggested a high need for hospitalization. Also, a score of 3 or more had a sensitivity of 87.5% (95% CI 95.9, 94.8) and a specificity of 84.0% (95% CI 80.2, 87.4) for hospitalization.

DISCUSSION

This study demonstrates that in an area of high immunisation uptake that the prevalence of bacterial pneumonia in children presenting in primary care with clinically defined pneumonia is low. The introduction of both Haemophilus influenzae type b and pneumococcal conjugate vaccines has been shown to reduce

the incidence of severe bacterial infections and hospitalization among children.[15,16] This data is consistent with the high uptake of immunisations in our cohort. A recent study has also shown that, in Malawi, the number of children with non-severe, fast-breathing pneumonia that needed amoxicillin treatment, for one child to benefit was 33[17] which is supportive of our findings also. The number of children with bacterial pneumonia has declined significantly since a previous study in the community in Eastern Africa performed prior to the introduction of pneumococcal vaccine[6] although, this used different microbiological techniques to assign diagnoses. The PERCH study which examined the causes of severe childhood pneumonia requiring hospital admission has also shown a predominantly viral aetiology even in this severe subset[18]. Overall, very few studies have been conducted in primary care in the era prior to the introduction of pneumococcal vaccine[19]. Given that most children with pneumonia will present initially to primary care, there is a clear need for further evaluation of both the aetiology and severity profile of childhood pneumonia in this setting.

The markers of severity (defined as hospitalization) in the BIOTOPE study are different from that outlined in current WHO guidelines even those these are the guidelines used to guide who should be referred to hospital. Our study shows that WHO severity criteria are present in a minority (30.4%) of children hospitalised, consistent with recent work[20] showing that 39% of fatal cases of pneumonia were defined as having non-severe pneumonia requiring only home treatment by the 2013 revision. The fact that the WHO criteria would have discharged these children with oral antibiotics

For peer review only - http://bmjopen.bmj.com/site/about/guidelines.xhtml

highlights the need for new markers of severity, as these guidelines are used to guide primary care workers in their decision-making regarding escalation of care. Our study highlights the changing pattern of markers of severity for hospitalization and generates new markers of severity that could be used in the community. We present a new 7-point clinical prediction rule (DIFFICULTY DRAWING Breath score) based on independent predictors of severity with a high predictive power for severity, defined as need for hospitalization. This will need to be evaluated in future studies.

This study involves an urban centre in an area of high immunisation and results may differ in different settings, particularly in locations with low immunisation uptake rates or where healthcare may not be easily accessed e.g. remote areas. Infection rates for both respiratory viruses and malaria are subject to significant seasonal variation, as such, had the study been conducted during an alternative time period or in an alternative geographical location, the results may have differed significantly. However, given the study site was representative of Malawi and the Mzuzu region as a whole in terms of immunisation rates, we believe our findings may be generalised and applied to Malawi as a nation and (to a certain extent) other nations of sub-Saharan Africa. More studies are required in different epidemiologic settings to generate further data. The lack of a control group (i.e., well children) limited our ability to verify the clinical significance of most viral pathogens and atypical bacteria. However, all children were included based on a clinical definition of pneumonia and therefore had pre-existing illness signs. Our rate of identification of a possible

For peer review only - http://bmjopen.bmj.com/site/about/guidelines.xhtml

For peer review only

pathogen was high and this likely reflects the use of molecular techniques. The lack of chest radiography may be seen as a limitation but previous studies have shown it is not predictive of aetiology and does not improve outcomes[22]. Also, plain film radiographs would not be routinely available in primary care.

Our findings indicate that among pre-school aged children in a region with high rates of immunisation, a high percentage of WHO clinically defined pneumonia are likely to be of viral origin. It is known that respiratory viruses, including rhinoviruses, can cause severe lower respiratory tract disease, particularly in infants and young children[23,24]. There is also a change in the clinical markers of severe illness, as defined by hospitalization. The current WHO markers of severity have a low sensitivity for hospitalization. This has important implications for the management of childhood pneumonia in the community and may reflect the change in clinical signs in the era of high immunisation uptake.

Conclusion:

In Mzuzu, Malawi, the vast majority of children with a WHO clinical definition of pneumonia had a viral illness and thus did not require an antibiotic. Current guidelines suggest all of these children should receive antibiotics. This subjects the

For peer review only - http://bmjopen.bmj.com/site/about/guidelines.xhtml

community to accelerated development of antimicrobial resistance. The current definition of severe clinical signs used by the WHO has a poor sensitivity and this is potentially attributable to the high uptake of immunisations against respiratory bacteria in this cohort, with a corresponding evolution of disease presentation. Further study is warranted to determine if a revision of current WHO guidelines is required, thereby ensuring children are appropriately are referred to hospital. The score presented here provides a practical alternative, should it be more widely validated.

For peer review only

Table 1 Baseline demographic and clinical characteristics

	Total Population	Not hospitalised	Hospitalised
**Demographics**	**N=494**	**(n=438)**	**(n=56)**

Age (months)	Median [IQR]	18 [10-30]	19 [10-32]	13 [6-21]	<0.001
Age 2 - 11 months	n (%)	157/494 (31.8%)	132/438 (30.1%)	25/56 (44.6%)	0.033
Age 12 - 35 months	n (%)	252/494 (51.0%)	224/438 (51.1%)	28/56 (50.0%)	NS
Age 36 - 60 months	n (%)	85/494 (17.2%)	82/438 (18.7%)	3/56 (5.3%)	0.013
Male	n (%)	271/494 (54.8%)	240/438 (54.8%)	31/56 (55.4%)	NS
Weight for age[1] > -1 SD	n (%)	371/481 (77.1%)	336/438 (76.7%)	35/56 (62.5%)	0.032
Weight for age -2 to -1 SD	n (%)	68/481 (14.1%)	55/438 (12.6%)	13/56 (23.2%)	0.038
Weight for age -3 to -2 SD	n (%)	34/481 (7.1%)	27/438 (6.1%)	7/56 (12.5%)	NS
Weight for age < -3 SD	n (%)	8/481 (1.7%)	8/438 (1.8%)	0/56 (0%)	NS
Respiratory rate (bpm)	Median [IQR]	60 [54-65]	59 [52.5-64]	68 [62-72]	<0.0001
Body temperature (°C)	Median [IQR]	37.4 [36.8-38.2]	37.4 [36.8-38.1]	37.8 [37.1-38.6]	0.015
Antibiotics given	n (%)	442/466 (94.8%)	390/411 (94.9%)	52/55 (94.6%)	NS
Antimalarial given	n (%)	41/441 (9.3%)	34/392 (8.7%)	7/49 (14.3%)	NS
HIV positive[3]	n (%)	11/494 (2.2%)	10/438 (2.2%)	1/56 (1.8%)	NS

[1] Weight was not recorded in 11 children and 3 children were oedematous.

For peer review only - http://bmjopen.bmj.com/site/about/guidelines.xhtml

For peer review only

Malaria RDT positive	n (%)	70/485 (14.3%)	59/438 (13.5%)	11/56 (19.6%)	NS
**Clinical Characteristics**					
WHO defined severity[2]	n (%)	64/494 (12.9%)	47/438 (10.7%)	17/56 (30.4%)	<0.001
Fever	n (%)	470/494 (95.1%)	417/438 (95.2%)	53/56 (94.6%)	NS
Cough	n (%)	492/494 (99.6%)	436/438 (99.5%)	56/56 (100%)	NS
Vomiting	n (%)	122/494 (24.7%)	99/438 (22.6%)	23/56 (41.1%)	0.005
Diarrhoea	n (%)	22/494 (4.5%)	19/438 (4.3%)	3/56 (5.4%)	NS
Abnormal sleepiness	n (%)	8/494 (1.7%)	6/438 (1.4%)	2/56 (3.6%)	NS
Coryza	n (%)	259/494 (52.4%)	230/438 (52.5%)	29/56 (51.9%)	NS
Sneezing	n (%)	156/494 (31.6%)	144/438 (32.9%)	12/56 (21.4%)	NS
Difficulty breathing	n (%)	184/494 (37.2%)	138/438 (31.5%)	46/56 (82.1%)	<0.0001
Deep breathing	n (%)	53/494 (34.8%)	26/438 (5.9%)	27/56 (48.2%)	<0.0001

[2] Describes 64 children with any one of severe malnutrition (weight for age < 3SD, n=8), unable to feed/drink (n=50), stridor (n=9), lethargy (n=0), unconsciousness (n=0), vomiting everything (n=0), convulsions (n=0).

[3] 184 consented to testing

For peer review only - http://bmjopen.bmj.com/site/about/guidelines.xhtml

Wheeze		n (%)	36/494 (7.3%)	25/438 (5.7%)	11/56 (19.6%)	0.001
In-drawing lower chest wall		n (%)	172/494 (34.8%)	128/438 (29.2%)	44/56 (78.6%)	<0.0001
Nasal flaring		n (%)	216/494 (43.7%)	175/438 (40.0%)	41/56 (73.2%)	<0.0001
Grunting		n (%)	28/494 (5.6%)	14/438 (3.2%)	14/56 (25.0%)	<0.0001
Head nodding		n (%)	17/494 (3.4%)	10/438 (2.2%)	7/56 (12.5%)	0.001

Abbreviations: bpm, breaths per minute; C, Celsius; HIV human immunodeficiency virus; RDT, rapid diagnostic test.

Table 2: Microbiologic analyses

For peer review only - http://bmjopen.bmj.com/site/about/guidelines.xhtml

		Total Population	Not hospitalised	Hospitalised	
**Bacterial**[3]		**N=488**	**(n=432)**	**(n=56)**	
S. pneumoniae	n (%)	4/494 (0.8%)	4/438 (0.9%)	0/56 (0 %)	NS
S. aureus	n (%)	2/494 (0.4%)	2/438 (0.5%)	0/56 (0%)	NS
Haemophilus spp.	n (%)	2/494 (0.4%)	2/438 (0.5%)	0/56 (0%)	NS
M. pneumoniae	n (%)	3/494 (0.6%)	3/438 (0.7%)	0/56 (0%)	NS
C._pneumoniae	n (%)	2/494 (0.4%)	2/438 (0.5%)	0/56 (0%)	NS
Any bacterial infection	n (%)	13/494 (2.6%)	13/438 (3.0%)	0/56 (0%)	NS
**Viral**[4]					
Adenovirus	n (%)	82/494 (16.6%)	71/438 (16.2%)	11/56 (19.6%)	NS
Bocavirus	n (%)	100/494 (20.2%)	92/438 (21%)	8/56 (20.5%)	NS
CoV* HKU	n (%)	0/494 (0%)	0/438 (0%)	0/56 (0%)	NS
CoV* NL63	n (%)	1/494 (0.2%)	1/438 (0.2%)	0/56 (0%)	NS
CoV* 229E	n (%)	1/494 (0.2%)	1/438 (0.2%)	0/56 (0%)	NS
CoV* OC43	n (%)	1/494 (0.2%)	1/438 (0.2%)	0/56 (0%)	NS
Influenza A	n (%)	76/494 (15.4%)	74/438 (16.9%)	2/56 (15.6%)	NS
Influenza AH1	n (%)	64/494 (13%)	63/438(14.4%)	1/56 (1.8%)	0.001
Influenza AH3	n (%)	6/494 (1.2%)	6/438 (1.4%)	0/56 (0%)	NS

[3] Two other bacterial species were identified in the patients who were not hospitalized, but were not clinically associated with pneumonia (E. Coli, and Shigella).
[4] In 6 cases it was not possible to obtain a valid PCR run

For peer review only - http://bmjopen.bmj.com/site/about/guidelines.xhtml

Influenza B	n (%)	9/494 (1.8%)	8/438 (1.8%)	1/56 (1.8%)	NS
HMPV+	n (%)	73/494 (14.7%)	63/438 (14.4%)	10/56 (15.0%)	NS
Parainfluenza 1	n (%)	11/494 (2.2%)	10/438 (2.3%)	1/56 (1.8%)	NS
Parainfluenza 2	n (%)	10/494 (2.0%)	10/438 (2.3%)	0/56 (0%)	NS
Parainfluenza 3	n (%)	4/494 (0.8%)	3/438 (0.7%)	1/56 (1.8%)	NS
Parainfluenza 4	n (%)	17/494 (3.4%)	15/438 (3.4%)	2/56 (3.6%)	NS
RSV// A	n (%)	164/494 (33.2%)	144/438 (32.9%)	20/56 (35.7%)	NS
RSV// B	n (%)	50/488 (10.1%)	44/438(10%)	6/56 (10.7%)	NS
Rhino/enterovirus	n (%)	295/494 (59.7%)	263/438 (60%)	32/56 (57.1%)	NS
Any virus	n (%)	940/488448/494 (93.1%)	409/432 (94.7%)	51/56 (91.1%)	NS

* = Coronavirus

+ = Human metapneumovirus

// = Respiratory syncytial virus

For peer review

For peer review only - http://bmjopen.bmj.com/site/about/guidelines.xhtml

Table 3: Sensitivity and Specificity of WHO Markers of Severe Pneumonia for hospitalization

Variable	Sensitivity (CI 95%)	Specificity (CI 95%)
Severe pneumonia	0.304 (0.188, 0.441)	0.9 (0.868, 0.926)
Vomiting everything	-	0.996 (0.984, 0.999)
Convulsions	-	1 (0.992, 1.000)
Lethargy	-	1 (0.992, 1.000)
Unconsciousness	-	1 (0.992, 1.000)
Malnourished	-	0.987 (0.971, 0.995)
Unable to feed	0.214 (0.116, 0.344)	0.916 (0.886, 0.940)
Stridor	0.089 (0.030, 0.196)	0.991 (0.977, 0.998)
Any sign	0.179 (0.089, 0.304)	0.938 (0.911, 0.958)

For peer review

For peer review only - http://bmjopen.bmj.com/site/about/guidelines.xhtml

Contributorship Statement:

Contributors JG and CW conceived the paper, assisted with study design and secured funding. ML performed data analysis, interpretation and drafted the manuscript. SD, RD, NG, CDG, MC, DW and JW assisted with study design, analysis and interpretation of data. All authors contributed to manuscript development, edited for critical content, and have approved the final version.

Competing Interest Statements:

The authors declare that there are no competing interests of any nature, financial or otherwise.

Funding:

This work was conducted with the generous support of the Bill & Melinda Gates Foundation – Investment ID: OPP1139557

Data Sharing Statement:

Study data pertaining to the BIOTOPE Cohort, may be made available upon reasonable request from the corresponding author.

For peer review only - http://bmjopen.bmj.com/site/about/guidelines.xhtml

References:

1. Liu L, Oza S, Hogan D, et al. Global, regional, and national causes of child mortality in 2000-13, with projections to inform post-2015 priorities: an updated systematic analysis. *Lancet.* 2015;385(9966):430-440.
2. Manenzhe RI, Zar HJ, Nicol MP, Kaba M. The spread of carbapenemase-producing bacteria in Africa: a systematic review. *J Antimicrob Chemother.* 2015;70(1):23-40.
3. Carattoli A. Plasmids and the spread of resistance. *Int J Med Microbiol.* 2013;303(6-7):298-304.
4. Baker KS, Dallman TJ, Ashton PM, et al. Intercontinental dissemination of azithromycin-resistant shigellosis through sexual transmission: a cross-sectional study. *The Lancet Infectious diseases.* 2015;15(8):913-921.
5. Prasad N, Murdoch DR, Reyburn H, Crump JA. Etiology of Severe Febrile Illness in Low- and Middle-Income Countries: A Systematic Review. *PloS one.* 2015;10(6):e0127962.
6. D'Acremont V, Kilowoko M, Kyungu E, et al. Beyond malaria--causes of fever in outpatient Tanzanian children. *N Engl J Med.* 2014;370(9):809-817.
7. Mtove G, Hendriksen IC, Amos B, et al. Treatment guided by rapid diagnostic tests for malaria in Tanzanian children: safety and alternative bacterial diagnoses. *Malar J.* 2011;10:290.
8. Brent AJ, Ahmed I, Ndiritu M, et al. Incidence of clinically significant bacteraemia in children who present to hospital in Kenya: community-based observational study. *Lancet.* 2006;367(9509):482-488.
9. Hopkins H, Bruxvoort KJ, Cairns ME, et al. Impact of introduction of rapid diagnostic tests for malaria on antibiotic prescribing: analysis of observational and randomised studies in public and private healthcare settings. *BMJ (Clinical research ed).* 2017;356:j1054.
10. Hammitt LL, Kazungu S, Morpeth SC, et al. A preliminary study of pneumonia etiology among hospitalized children in Kenya. *Clin Infect Dis.* 2012;54 Suppl 2:S190-199.
11. . *Revised WHO Classification and Treatment of Pneumonia in Children at Health Facilities: Evidence Summaries*. Geneva2014.
12. Das S, Bryan K, Buckley PG, et al. Modulation of neuroblastoma disease pathogenesis by an extensive network of epigenetically regulated microRNAs. *Oncogene.* 2013;32(24):2927-2936.
13. Watson CJ, Gupta SK, O'Connell E, et al. MicroRNA signatures differentiate preserved from reduced ejection fraction heart failure. *European journal of heart failure.* 2015;17(4):405-415.
14. Harris PA, Taylor R, Thielke R, Payne J, Gonzalez N, Conde JG. Research electronic data capture (REDCap)--a metadata-driven methodology and workflow process for providing translational research informatics support. *Journal of biomedical informatics.* 2009;42(2):377-381.
15. Fitzwater SP, Chandran A, Santosham M, Johnson HL. The worldwide impact of the seven-valent pneumococcal conjugate vaccine. *Pediatr Infect Dis J.* 2012;31(5):501-508.

For peer review only

16. Theodoratou E, Johnson S, Jhass A, et al. The effect of Haemophilus influenzae type b and pneumococcal conjugate vaccines on childhood pneumonia incidence, severe morbidity and mortality. *Int J Epidemiol.* 2010;39 Suppl 1:i172-185.
17. Ginsburg AS, Mvalo T, Nkwopara E, et al. Placebo vs Amoxicillin for Nonsevere Fast-Breathing Pneumonia in Malawian Children Aged 2 to 59 Months: A Double-blind, Randomized Clinical Noninferiority Trial. *JAMA Pediatr.* 2018.
18. Pneumonia Etiology Research for Child Health Study G. Causes of severe pneumonia requiring hospital admission in children without HIV infection from Africa and Asia: the PERCH multi-country case-control study. *Lancet.* 2019.
19. Gilani Z, Kwong YD, Levine OS, et al. A literature review and survey of childhood pneumonia etiology studies: 2000-2010. *Clin Infect Dis.* 2012;54 Suppl 2:S102-108.
20. Agweyu A, Lilford RJ, English M, Clinical Information Network Author G. Appropriateness of clinical severity classification of new WHO childhood pneumonia guidance: a multi-hospital, retrospective, cohort study. *The Lancet Global health.* 2018;6(1):e74-e83.
21. Madhi SA, Klugman KP, Vaccine Trialist G. A role for Streptococcus pneumoniae in virus-associated pneumonia. *Nat Med.* 2004;10(8):811-813.
22. Harris M, Clark J, Coote N, et al. British Thoracic Society guidelines for the management of community acquired pneumonia in children: update 2011. *Thorax.* 2011;66 Suppl 2:ii1-23.
23. Jain S, Self WH, Wunderink RG, et al. Community-Acquired Pneumonia Requiring Hospitalization among U.S. Adults. *N Engl J Med.* 2015;373(5):415-427.
24. Costa LF, Queiroz DA, Lopes da Silveira H, et al. Human rhinovirus and disease severity in children. *Pediatrics.* 2014;133(2):e312-321.

For peer review only

For peer review only - http://bmjopen.bmj.com/site/about/guidelines.xhtml

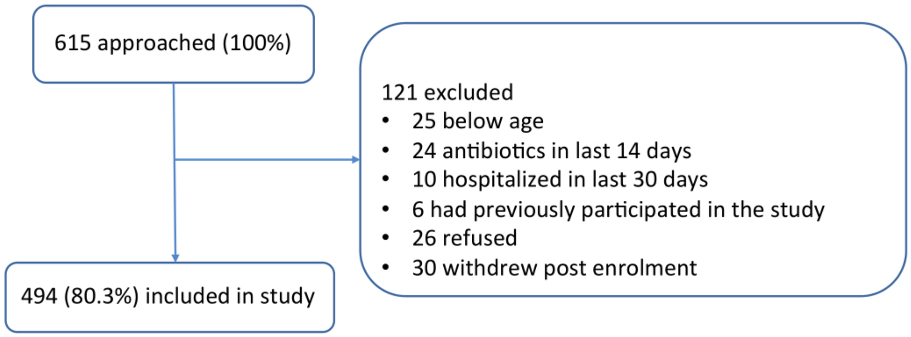

402x165mm (72 x 72 DPI)

BMJ Open

Aetiology and Severity of Childhood Pneumonia in Primary Care in Malawi: A Cohort Study

Journal:	*BMJ Open*
Manuscript ID	bmjopen-2020-046633.R1
Article Type:	Original research
Date Submitted by the Author:	05-Apr-2021
Complete List of Authors:	Gallagher, Joe; University College Dublin College of Health Sciences, gHealth Research Institute<br>Chisale, Master; Mzuzu Central Hospital<br>Das, Sudipto; Royal College of Surgeons in Ireland<br>Drew, Richard; Irish Meningitis and Sepsis Reference Laboratory<br>Gleseva, Nadezhda; University College Dublin College of Health Sciences, gHealth Research Institute<br>Wildes, Dermot; University College Dublin College of Health Sciences, gHealth Research Institute<br>De Gascun, Cillian; National Virus Reference Laboratory<br>Wu, Tsung-Shu; Luke International Norway; Overseas Mission Department, Pingtung Christian Hospital<br>Ledwidge, Mark; University College Dublin College of Health Sciences, gHealth Research Institute<br>Watson, Chris; Queen's University Belfast Wellcome-Wolfson Institute for Experimental Medicine
**Primary Subject Heading**:	Paediatrics
Secondary Subject Heading:	Global health, Evidence based practice, Public health
Keywords:	Epidemiology < TROPICAL MEDICINE, Microbiology < PATHOLOGY, Paediatric infectious disease & immunisation < PAEDIATRICS, Paediatric thoracic medicine < PAEDIATRICS, PRIMARY CARE

SCHOLARONE™
Manuscripts

I, the Submitting Author has the right to grant and does grant on behalf of all authors of the Work (as defined in the below author licence), an exclusive licence and/or a non-exclusive licence for contributions from authors who are: i) UK Crown employees; ii) where BMJ has agreed a CC-BY licence shall apply, and/or iii) in accordance with the terms applicable for US Federal Government officers or employees acting as part of their official duties; on a worldwide, perpetual, irrevocable, royalty-free basis to BMJ Publishing Group Ltd ("BMJ") its licensees and where the relevant Journal is co-owned by BMJ to the co-owners of the Journal, to publish the Work in this journal and any other BMJ products and to exploit all rights, as set out in our licence.

The Submitting Author accepts and understands that any supply made under these terms is made by BMJ to the Submitting Author unless you are acting as an employee on behalf of your employer or a postgraduate student of an affiliated institution which is paying any applicable article publishing charge ("APC") for Open Access articles. Where the Submitting Author wishes to make the Work available on an Open Access basis (and intends to pay the relevant APC), the terms of reuse of such Open Access shall be governed by a Creative Commons licence – details of these licences and which Creative Commons licence will apply to this Work are set out in our licence referred to above.

Other than as permitted in any relevant BMJ Author's Self Archiving Policies, I confirm this Work has not been accepted for publication elsewhere, is not being considered for publication elsewhere and does not duplicate material already published. I confirm all authors consent to publication of this Work and authorise the granting of this licence.

For peer review only

Aetiology and Severity of Childhood Pneumonia in Primary Care in Malawi: A Cohort Study

Joe Gallagher, MD[1*], Master Chisale, MSc[1,2], Sudipto Das, PhD[3], Richard Drew, MD[3,4], Nadezhda Glezeva, PhD[1], Dermot M Wildes[1], MB, Cillian De Gascun, MD[5], Tsung-Shu Joseph Wu, MSc[6,7], Mark Ledwidge, PhD[1], Chris J Watson, PhD[1,8].

On behalf of the BIOTOPE team

[1]gHealth Research Group, UCD Conway Institute, School of Medicine, University College Dublin, Ireland.

[2]Mzuzu Central Hospital, Mzuzu, Malawi.

[3]Royal College of Surgeons in Ireland, Dublin, Ireland.

[4] Irish Meningitis and Sepsis Reference Laboratory, Temple Street, Dublin 2.

[5] UCD National Virus Reference Laboratory, University College Dublin, Ireland.

[6] Luke International Norway, Mzuzu, Malawi.

[7]Overseas Mission Department, Pingtung Christian Hospital, Taiwan.

[8] Wellcome-Wolfson Institute for Experimental Medicine, Queen's University Belfast, Northern Ireland.

For peer review only - http://bmjopen.bmj.com/site/about/guidelines.xhtml

For peer review only

* Corresponding author:

Dr. Joe Gallagher,

School of Medicine,

University College Dublin,

Dublin.

Tel: 053 9421446

Fax: 053 9422526

Email: joejgallagher@gmail.com

For peer review only

Manuscript word count: 2,358 (Excluding Abstract, Tables, Figures & References)

Ethics - The protocol and related documents were successfully vetted by the National Health Science Research Committee of Malawi - Ethics approval number 15/11/1532

For peer review only - http://bmjopen.bmj.com/site/about/guidelines.xhtml

Abstract

Objective:

To determine the aetiology of community acquired pneumonia in children presenting to primary care in Northern Malawi, and to ascertain predictors for identification of children requiring hospitalisation.

Design: The BIOmarkers TO diagnose PnEumonia (BIOTOPE) study was a prospective cohort study conducted from March to June 2016.

Setting: Primary care in Northern Malawi.

Patients: 494 children aged 2 months to 59 months with World Health Organization defined pneumonia.

Main Outcome(s) and Measure(s): Number of children with bacterial infection identified and the sensitivity/specificity of WHO markers of severity for need for hospitalisation.

Results:

13 (2.6%) children had a bacterium consistent with pneumonia identified. A virus consistent with pneumonia was identified in in 448 (90.7%) of children. 56 children were admitted to hospital and two children died within 30 days. 442 (89.5%) received antibiotic therapy. Eleven children (2.6%) had HIV. WHO severity markers at baseline demonstrated poor sensitivity for the need for hospitalization with a sensitivity of 0.303 (95% confidence interval 0.188-0.441) and a specificity 0.9 (95% confidence

For peer review only

interval 0.868 to 0.926). A prediction rule to indicate the need for hospitalization was developed.

Conclusions and Relevance:

The low rate of bacterial infection and high use of antibiotics in the setting of high immunisation rates highlights the changing profile of childhood pneumonia. Similarly, the markers of need for hospitalization may have changed in the setting of extended immunisation. Further studies are required to examine this.

Article Summary:

- The pneumococcal vaccine was introduced in 2012 in Malawi and this study provides data on the aetiology and severity markers in primary care following the introduction of this vaccine.
- It demonstrates the significant reduction in bacterial infection compared to the previous studies undertaken using similar methods prior to introduction of this vaccine in Africa
- It also highlights that the severity markers in childhood pneumonia are changing and this is important since these markers are still used in primary care in Africa for the determination of referral to hospital.
- The study was in an urban area with high immunisation rates over one season and the lack of a control group (i.e., well children) limited our ability to verify the clinical significance of most viral pathogens and atypical bacteria

For peer review

For peer review only - http://bmjopen.bmj.com/site/about/guidelines.xhtml

INTRODUCTION

Despite recent advances in reducing childhood mortality, pneumonia remains a major cause of morbidity and mortality among children aged under five years, with an estimated 935,000 deaths per annum[1]. Allied to this is the growing concern regarding antimicrobial resistance driven by overuse of antibiotics[2, 3] and intercontinental spread of this resistance[4]. The increased use of universal immunisation, including haemophilus influenzae type B (Hib) and pneumococcal vaccines are likely to have altered the proportion with viral illness. This may also have altered the aetiology, severity, and host response in childhood pneumonia. A greater understanding and characterisation of these factors in primary care is required to continue advances in reducing childhood mortality from pneumonia.

Current strategies for the management of childhood pneumonia in developing countries focus on the identification of the illness and provision of antibiotics, despite the low reported prevalence of bacterial infection[6-9]. Most studies have focused on the hospitalised population, and yet in developing countries, it is in the community where these patients are most likely to present. Therefore, contemporary estimates of the microbiological causes of pneumonia and predictors of hospitalization would be of benefit. The use of microbiological techniques is hampered by the high rate of carriage of bacteria and viruses in children[10]. Finally, WHO markers of severity, which are used to guide referral for hospitalization in primary care, are mostly based on clinical symptoms alone and advise the use of pulse oximetry to help determine severity if available. However oximeters are rarely available in primary care in countries such as Malawi[11].

For peer review only - http://bmjopen.bmj.com/site/about/guidelines.xhtml

The BIOTOPE study (BIOmarkers TO diagnose PnEumonia) was a prospective cohort study and this paper reports on the aetiology of community acquired pneumonia in children presenting in primary care in Northern Malawi and predictors of children requiring hospitalization.

METHODS

Study Sites & Population

From March to June 2016, we recruited children at Mapale Health Centre and the outpatient department of Mzuzu Central Hospital in Mzuzu, Northern Malawi – both of which serve as primary care facilities for this urban area. Shortly after study commencement, the outpatient department of Mzuzu Central Hospital ceased to provide primary care services. As such, 98% of participants were recruited from Mapale Health Centre. Mapale Health Centre currently serves the whole city as a primary health care facility (population of approximately 220,000) The under-five population in this setting is characterised by a high burden of infectious disease including acute respiratory infections (ARI), diarrhoea, and malaria. In 2016 the estimated coverage was 83% for pneumococcal conjugate vaccine and 84% for Haemophilus influenza type B vaccine in Malawi,[12] (Further details on site are available in the supplemental file)

Study participants:

For peer review only - http://bmjopen.bmj.com/site/about/guidelines.xhtml

Children presenting to the primary care facilities were assessed for study enrolment. Children aged 2-59 months were deemed eligible to participate in the study if the main presenting complaint was cough or difficulty breathing associated with tachypnoea (defined as >50 breaths per minute if aged 2-11 months or > 40 breaths per minute age if aged 12-59 months) or chest in-drawing. This is the current WHO clinical case definition of pneumonia[9]. Exclusion criteria listed were as follows: those discharged from hospital in the preceding 30 days; those who had completed a course of antibiotics within 14 days of presentation; or those who had received antibiotics prior to clinical assessment for this illness. The study details were discussed with a parent/guardian of any patient who fit our inclusion criteria, who in turn provided informed consent in a written format. The protocol and related documents were approved by the National Health Science Research Committee of Malawi and the Mzuzu Central Hospital Research Committee. Patients received timely treatment and supportive care as required. The study was conducted in compliance with the ethical standards as outlined by two ethics committees and was aligned with the values outlined by the Declaration of Helsinki. (National Health Research Committee of Malawi Ethics Approval #15/11/1532.

For peer review only

Study Procedures

Clinical Assessment

In order to record clinical data, a standardized report form was provided to our investigators incorporating demographics, risk factors, and items relevant to a pneumonia presentation. Immunisation records are held by patients in a patient held record known as the “health passport”. Only those who had a health passport with

For peer review only - http://bmjopen.bmj.com/site/about/guidelines.xhtml

recorded immunisations were used for purpose of recording immunisation status. Where possible, children were followed up by contacting a caregiver at 7 days and 30 days following initial assessment by telephone. All children had a full blood count and malaria rapid diagnostic test. HIV rapid diagnostic testing was offered to all children aged over 12 months and HIV PCR testing to all children aged 2 months to 11 months.

Microbiologic and Other Investigations

For all patients, blood samples (8 ml) and pooled naso/oro-pharyngeal swabs were collected for microbiological investigation on site, and for further serologic and molecular analysis in Ireland. A systematic set of investigations were performed for all patients. Blood cultures were processed locally. Blood samples for PCR for *Streptococcus pnuemoniae*, *Staphylococcus aureus* and *Haemophilus influenzae* along with naso/oro-pharyngeal swabs for respiratory viruses and atypical bacteria were processed in Ireland. (Further details in the supplemental file)

Definitive Diagnosis Based on Predefined Criteria

The final diagnosis (in some cases, diagnoses) was ascertained for each patient using pre-determined criteria for both clinical and microbiological assessment. A case was deemed to be "severe" should the child's condition fulfil WHO criteria for this state. Bacterial pneumonia was diagnosed on a positive blood culture or positive blood PCR for a relevant organism or positive nasopharyngeal swab for C. pneumoniae or M. pneumonia. Laboratory data were combined with the predetermined clinical criteria to establish each diagnosis.

For peer review only - http://bmjopen.bmj.com/site/about/guidelines.xhtml

Statistical Analyses

The overall aim of this project was to determine blood and urine biomarkers of host response in bacterial pneumonia in a cohort of children age 2- 59 months in a single centre in Malawi. Based on the study by D'Acrement[6] et al it was estimated that the prevalence of bacterial infection among febrile children with pneumonia would be 20%. The required sample size for our study based on this prevalence was given by the formula $n = 4pq/d^2$ where p = prevalence q = 100-p and d= the precision of the estimate. Assuming a relative precision of 20% and based on previous pilot work on follow up and sample analysis this resulted in a required sample size of n= 490.

Data were entered into REDCap electronic data capture tools[13]. All statistical analyses were performed using R software (R Foundation for Statistical Computing). Sensitivity and specificity of WHO severity markers for hospitalization were calculated, with the Clopper-Pearson method used for their confidence intervals. Logistic regression using pre-defined variables was undertaken to identify a new clinical prediction model and the Area under the curve (AUC) was calculated by area summation, Tjur r-squared estimate and Hanley & McNeil's (1982) SE formula was used for AUC confidence intervals. A simplified clinical prediction rule was developed by selecting independent predictors of hospitalization measurable from multivariable analyses, allocating a single point for each characteristic and creating a sum score for each patient. We applied Receiver Operated Characteristic (ROC) analysis at various point thresholds of the score.

For peer review only - http://bmjopen.bmj.com/site/about/guidelines.xhtml

Patient & Public Involvement Statement

Patients were not involved in concept design, participant recruitment or study conduct at any stage from inception to completion of this project.

RESULTS

Study Sample

A total of 615 children were approached for inclusion in this study. (Figure 1). Demographic and clinical characteristics of the children included in the study are described in Table 1. At day seven, 225 caregivers were contactable by phone and 100% of children were alive. At day thirty, 195 caregivers were contactable and two children had died.

Immunisations

317 patients had a patient passport with immunisation record available. 239 of these patients were over the age of 12 months, and 109 were over 24 months of age. 98 (89.9%) of those aged 24 months or over who had immunisation records available had received full immunisations. Furthermore, of the 56 patients who were hospitalised, 45 had immunization records available. We age and sex matched this group in a 1:2 ratio with 90 patients who were not-hospitalised and had immunization records available. The median number of immunisations amongst the hospitalized cohort was significantly lower than the non-hospitalised cohort (9 [IQR 7,10] vs. 10 [IQR10,10], p=0.003). Children without full complement of vaccinations

For peer review only - http://bmjopen.bmj.com/site/about/guidelines.xhtml

required by age one year were more likely to be hospitalised (RR 5.5, [95% CI: 1.88-16.1], p=0.008) than those with the correct vaccinations in age and sex adjusted analyses.

Microbiological Analyses

A bacterium consistent with pneumonia was identified in 13 (2.6%) children. In three cases, more than one bacterial cause was identified in the same child. Respiratory viruses were found in 12 of the 13 children considered to have bacterial pneumonia. In 448 (90.7%) of children a respiratory virus alone was identified on testing. Full details of the microbiological analyses are provided in Table 2 and the supplemental file. In 33 (6.7%) cases, no organism associated with pneumonia was identified.

WHO Severity Criteria and Hospitalization

WHO severity signs and other variables were recorded prospectively. The sensitivity and specificity of these are in Table 3.

Using logistic regression, further models were developed with significant improvement in discriminant value. In multivariable modeling, the following seven variables were predictive of hospitalization: Difficulty breathing, Deep breathing, Respiratory rate, Age, Wheeze, Lower chest wall indrawing. The AUC of the fully adjusted model for prediction of hospitalization was equal to 0.92 (95% CI 0.90, 0.93, p<0.0001). To create a simplified version of this model, we assigned a score of 1 to

each sign or symptom (including categorical treatment of Age <2 years and Respiratory Rate >70 bpm) and generated a simple, 7 point "DIFFICULTY DRAWING breath" score for each patient, which predicted need for hospitalization with an AUC of 0.91 (95% CI 0.87 to 0.95, p<0.0001). A score of 0-1 was associated with low need for hospitalization, 2-3 was intermediate and a score of 4 or greater suggested a high need for hospitalization. Also, a score of 3 or more had a sensitivity of 87.5% (95% CI 95.9, 94.8) and a specificity of 84.0% (95% CI 80.2, 87.4) for hospitalization. (Supplemental file)

DISCUSSION

This study demonstrates that in an area of high immunisation uptake that the prevalence of bacterial pneumonia in children presenting in primary care with clinically defined pneumonia is low. The introduction of both Haemophilus influenzae type b and pneumococcal conjugate vaccines has been shown to reduce the incidence of severe bacterial infections and hospitalization among children.[14, 15] This data is consistent with the high uptake of immunisations in our cohort. A recent study has also shown that, in Malawi, the number of children with non-severe, fast-breathing pneumonia that needed amoxicillin treatment, for one child to benefit was 33[16] which is supportive of our findings also. The number of children with bacterial pneumonia has declined significantly since a previous study in the community in Eastern Africa performed prior to the introduction of pneumococcal vaccine[6] although, this used different microbiological techniques to assign diagnoses. The PERCH study which examined the causes of severe childhood pneumonia requiring hospital admission has also shown a predominantly viral aetiology even in this

For peer review only - http://bmjopen.bmj.com/site/about/guidelines.xhtml

severe subset[17]. Overall, very few studies have been conducted in primary care[1817161717] in the era prior to the introduction of pneumococcal vaccine[19]. Given that most children with pneumonia will present initially to primary care, there is a clear need for further evaluation of both the aetiology and severity profile of childhood pneumonia in this setting.

The markers of severity (defined as hospitalization) in the BIOTOPE study are different from that outlined in current WHO guidelines even those these are the guidelines used to guide who should be referred to hospital. Our study shows that WHO severity criteria are present in a minority (30.4%) of children hospitalised, consistent with recent work[19] showing that 39% of fatal cases of pneumonia were defined as having non-severe pneumonia requiring only home treatment by the 2013 revision. The fact that the WHO criteria would have discharged these children with oral antibiotics highlights the need for new markers of severity, as these guidelines are used to guide primary care workers in their decision-making regarding escalation of care. Our study highlights the changing pattern of markers of severity for hospitalization and generates new markers of severity that could be used in the community. We present a new 7-point clinical prediction rule (DIFFICULTY DRAWING Breath score) based on independent predictors of severity with a high predictive power for severity, defined as need for hospitalization. This will need to be evaluated in future studies.

For peer review only - http://bmjopen.bmj.com/site/about/guidelines.xhtml

This study involves an urban centre in an area of high immunisation and results may differ in different settings, particularly in locations with low immunisation uptake rates or where healthcare may not be easily accessed e.g. remote areas. Infection rates for both respiratory viruses and malaria are subject to significant seasonal variation, as such, had the study been conducted during an alternative time period or in an alternative geographical location, the results may have differed significantly. However, given the study site was representative of Malawi and the Mzuzu region as a whole in terms of immunisation rates, we believe our findings may be generalised and applied to Malawi as a nation and (to a certain extent) other nations of sub-Saharan Africa. More studies are required in different epidemiologic settings to generate further data. The lack of a control group (i.e., well children) limited our ability to verify the clinical significance of most viral pathogens and atypical bacteria. However, all children were included based on a clinical definition of pneumonia and therefore had pre-existing illness signs. Our rate of identification of a possible pathogen was high and this likely reflects the use of molecular techniques. The lack of chest radiography may be seen as a limitation but previous studies have shown it is not predictive of aetiology and does not improve outcomes[20]. Also, plain film radiographs would not be routinely available in primary care.

Our findings indicate that among pre-school aged children in a region with high rates of immunisation, a high percentage of WHO clinically defined pneumonia are likely to be of viral origin. It is known that respiratory viruses, including rhinoviruses, can cause severe lower respiratory tract disease, particularly in infants and young children[21,22]. There is also a change in the clinical markers of severe illness, as defined by

hospitalization. The current WHO markers of severity have a low sensitivity for hospitalization. This has important implications for the management of childhood pneumonia in the community and may reflect the change in clinical signs in the era of high immunisation uptake.

Conclusion:

In Mzuzu, Malawi, the vast majority of children with a WHO clinical definition of pneumonia had a viral illness and thus did not require an antibiotic. Current guidelines suggest all of these children should receive antibiotics. This subjects the community to accelerated development of antimicrobial resistance. The current definition of severe clinical signs used by the WHO has a poor sensitivity and this is potentially attributable to the high uptake of immunisations against respiratory bacteria in this cohort, with a corresponding evolution of disease presentation. Further study is warranted to determine if a revision of current WHO guidelines is required, thereby ensuring children are appropriately are referred to hospital. The score presented here provides a practical alternative, should it be more widely validated.

For peer review only

For peer review only

For peer review only - http://bmjopen.bmj.com/site/about/guidelines.xhtml

Table 1 Baseline demographic and clinical characteristics

**Demographics**		**Total Population N=494**	**Not hospitalised (n=438)**	**Hospitalised (n=56)**	
Age (months)	Median [IQR]	18 [10-30]	19 [10-32]	13 [6-21]	<0.001
Age 2 - 11 months	n (%)	157/494 (31.8%)	132/438 (30.1%)	25/56 (44.6%)	0.033
Age 12 - 35 months	n (%)	252/494 (51.0%)	224/438 (51.1%)	28/56 (50.0%)	NS
Age 36 - 60 months	n (%)	85/494 (17.2%)	82/438 (18.7%)	3/56 (5.3%)	0.013
Male	n (%)	271/494 (54.8%)	240/438 (54.8%)	31/56 (55.4%)	NS
Weight for age[1] > -1 SD	n (%)	371/481 (77.1%)	336/438 (76.7%)	35/56 (62.5%)	0.032
Weight for age -2 to -1 SD	n (%)	68/481 (14.1%)	55/438 (12.6%)	13/56 (23.2%)	0.038
Weight for age -3 to -2 SD	n (%)	34/481 (7.1%)	27/438 (6.1%)	7/56 (12.5%)	NS
Weight for age < -3 SD	n (%)	8/481 (1.7%)	8/438 (1.8%)	0/56 (0%)	NS
Respiratory rate (bpm)	Median [IQR]	60 [54-65]	59 [52.5-64]	68 [62-72]	<0.0001

[1] Weight was not recorded in 11 children and 3 children were oedematous.

For peer review only - http://bmjopen.bmj.com/site/about/guidelines.xhtml

Body temperature (°C)	Median [IQR]	37.4 [36.8-38.2]	37.4 [36.8-38.1]	37.8 [37.1-38.6]	0.015
Antibiotics given	n (%)	442/466 (94.8%)	390/411 (94.9%)	52/55 (94.6%)	NS
Antimalarial given	n (%)	41/441 (9.3%)	34/392 (8.7%)	7/49 (14.3%)	NS
HIV positive[3]	n (%)	11/494 (2.2%)	10/438 (2.2%)	1/56 (1.8%)	NS
Malaria RDT positive	n (%)	70/485 (14.3%)	59/438 (13.5%)	11/56 (19.6%)	NS
**Clinical Characteristics**					
WHO defined severity[2]	n (%)	64/494 (12.9%)	47/438 (10.7%)	17/56 (30.4%)	<0.001
Fever	n (%)	470/494 (95.1%)	417/438 (95.2%)	53/56 (94.6%)	NS
Cough	n (%)	492/494 (99.6%)	436/438 (99.5%)	56/56 (100%)	NS
Vomiting	n (%)	122/494 (24.7%)	99/438 (22.6%)	23/56 (41.1%)	0.005
Diarrhoea	n (%)	22/494 (4.5%)	19/438 (4.3%)	3/56 (5.4%)	NS
Abnormal sleepiness	n (%)	8/494 (1.7%)	6/438 (1.4%)	2/56 (3.6%)	NS
Coryza	n (%)	259/494 (52.4%)	230/438 (52.5%)	29/56 (51.9%)	NS

[2] Describes 64 children with any one of severe malnutrition (weight for age < 3SD, n=8), unable to feed/drink (n=50), stridor (n=9), lethargy (n=0), unconsciousness (n=0), vomiting everything (n=0), convulsions (n=0).
[3] 184 consented to testing

For peer review only - http://bmjopen.bmj.com/site/about/guidelines.xhtml

Sneezing	n (%)	156/494 (31.6%)	144/438 (32.9%)	12/56 (21.4%)	NS
Difficulty breathing	n (%)	184/494 (37.2%)	138/438 (31.5%)	46/56 (82.1%)	<0.0001
Deep breathing	n (%)	53/494 (34.8%)	26/438 (5.9%)	27/56 (48.2%)	<0.0001
Wheeze	n (%)	36/494 (7.3%)	25/438 (5.7%)	11/56 (19.6%)	0.001
In-drawing lower chest wall	n (%)	172/494 (34.8%)	128/438 (29.2%)	44/56 (78.6%)	<0.0001
Nasal flaring	n (%)	216/494 (43.7%)	175/438 (40.0%)	41/56 (73.2%)	<0.0001
Grunting	n (%)	28/494 (5.6%)	14/438 (3.2%)	14/56 (25.0%)	<0.0001
Head nodding	n (%)	17/494 (3.4%)	10/438 (2.2%)	7/56 (12.5%)	0.001

Abbreviations: bpm, breaths per minute; C, Celsius; HIV human immunodeficiency virus; RDT, rapid diagnostic test.

For peer review only

Table 2: Microbiologic analyses

		Total Population	Not hospitalised	Hospitalised	
**Bacterial**[3]		**N=488**	**(n=432)**	**(n=56)**	
S. pneumoniae	n (%)	4/494 (0.8%)	4/438 (0.9%)	0/56 (0 %)	NS
S. aureus	n (%)	2/494 (0.4%)	2/438 (0.5%)	0/56 (0%)	NS
Haemophilus spp.	n (%)	2/494 (0.4%)	2/438 (0.5%)	0/56 (0%)	NS
M. pneumoniae	n (%)	3/494 (0.6%)	3/438 (0.7%)	0/56 (0%)	NS
C._pneumoniae	n (%)	2/494 (0.4%)	2/438 (0.5%)	0/56 (0%)	NS
Any bacterial infection	n (%)	13/494 (2.6%)	13/438 (3.0%)	0/56 (0%)	NS
**Viral**[4]					
Adenovirus	n (%)	82/494 (16.6%)	71/438 (16.2%)	11/56 (19.6%)	NS
Bocavirus	n (%)	100/494 (20.2%)	92/438 (21%)	8/56 (20.5%)	NS
CoV* HKU	n (%)	0/494 (0%)	0/438 (0%)	0/56 (0%)	NS
CoV* NL63	n (%)	1/494 (0.2%)	1/438 (0.2%)	0/56 (0%)	NS
CoV* 229E	n (%)	1/494 (0.2%)	1/438 (0.2%)	0/56 (0%)	NS
CoV* OC43	n (%)	1/494 (0.2%)	1/438 (0.2%)	0/56 (0%)	NS
Influenza A	n (%)	76/494 (15.4%)	74/438 (16.9%)	2/56 (15.6%)	NS

[3] Two other bacterial species were identified in the patients who were not hospitalized, but were not clinically associated with pneumonia (E. Coli, and Shigella).
[4] In 6 cases it was not possible to obtain a valid PCR run

For peer review only - http://bmjopen.bmj.com/site/about/guidelines.xhtml

					0.00
Influenza AH1	n (%)	64/494 (13%)	63/438(14.4%)	1/56 (1.8%)	1
Influenza AH3	n (%)	6/494 (1.2%)	6/438 (1.4%)	0/56 (0%)	NS
Influenza B	n (%)	9/494 (1.8%)	8/438 (1.8%)	1/56 (1.8%)	NS
HMPV[+]	n (%)	73/494 (14.7%)	63/438 (14.4%)	10/56 (15.0%)	NS
Parainfluenza 1	n (%)	11/494 (2.2%)	10/438 (2.3%)	1/56 (1.8%)	NS
Parainfluenza 2	n (%)	10/494 (2.0%)	10/438 (2.3%)	0/56 (0%)	NS
Parainfluenza 3	n (%)	4/494 (0.8%)	3/438 (0.7%)	1/56 (1.8%)	NS
Parainfluenza 4	n (%)	17/494 (3.4%)	15/438 (3.4%)	2/56 (3.6%)	NS
RSV[//] A	n (%)	164/494 (33.2%)	144/438 (32.9%)	20/56 (35.7%)	NS
RSV[//] B	n (%)	50/488 (10.1%)	44/438(10%)	6/56 (10.7%)	NS
Rhino/enterovirus	n (%)	295/494 (59.7%)	263/438 (60%)	32/56 (57.1%)	NS
Any virus	n (%)	940/488448/ 494 (93.1%)	409/432 (94.7%)	51/56 (91.1%)	NS

* = Coronavirus

\+ = Human metapneumovirus

// = Respiratory syncytial virus

For peer review only

Table 3: Sensitivity and Specificity of WHO Markers of Severe Pneumonia for hospitalization

Variable	Sensitivity (CI 95%)	Specificity (CI 95%)
Severe pneumonia	0.304 (0.188, 0.441)	0.9 (0.868, 0.926)
Vomiting everything	-	0.996 (0.984, 0.999)
Convulsions	-	1 (0.992, 1.000)
Lethargy	-	1 (0.992, 1.000)
Unconsciousness	-	1 (0.992, 1.000)
Malnourished	-	0.987 (0.971, 0.995)
Unable to feed	0.214 (0.116, 0.344)	0.916 (0.886, 0.940)
Stridor	0.089 (0.030, 0.196)	0.991 (0.977, 0.998)
Any sign	0.179 (0.089, 0.304)	0.938 (0.911, 0.958)

For peer review only - http://bmjopen.bmj.com/site/about/guidelines.xhtml

Contributorship Statement:

Contributors JG and CW conceived the paper, assisted with study design and secured funding. ML performed data analysis, interpretation and drafted the manuscript. SD, RD, NG, CDG, MC, DW and TSJW assisted with study design, analysis and interpretation of data. All authors contributed to manuscript development, edited for critical content, and have approved the final version.

Competing Interest Statements:

The authors declare that there are no competing interests of any nature, financial or otherwise.

Funding:

This work was conducted with the generous support of the Bill & Melinda Gates Foundation – Investment ID: OPP1139557

Data Sharing Statement:

Study data pertaining to the BIOTOPE Cohort, may be made available upon reasonable request from the corresponding author.

For peer review only - http://bmjopen.bmj.com/site/about/guidelines.xhtml

References:

1. Liu L, Oza S, Hogan D, et al. Global, regional, and national causes of child mortality in 2000-13, with projections to inform post-2015 priorities: an updated systematic analysis. *Lancet*. Jan 31 2015;385(9966):430-40. doi:10.1016/S0140-6736(14)61698-6
2. Manenzhe RI, Zar HJ, Nicol MP, Kaba M. The spread of carbapenemase-producing bacteria in Africa: a systematic review. *J Antimicrob Chemother*. Jan 2015;70(1):23-40. doi:10.1093/jac/dku356
3. Carattoli A. Plasmids and the spread of resistance. *Int J Med Microbiol*. Aug 2013;303(6-7):298-304. doi:10.1016/j.ijmm.2013.02.001
4. Baker KS, Dallman TJ, Ashton PM, et al. Intercontinental dissemination of azithromycin-resistant shigellosis through sexual transmission: a cross-sectional study. *The Lancet Infectious diseases*. Aug 2015;15(8):913-21. doi:10.1016/S1473-3099(15)00002-X
5. Prasad N, Murdoch DR, Reyburn H, Crump JA. Etiology of Severe Febrile Illness in Low- and Middle-Income Countries: A Systematic Review. *PloS one*. 2015;10(6):e0127962. doi:10.1371/journal.pone.0127962
6. D'Acremont V, Kilowoko M, Kyungu E, et al. Beyond malaria--causes of fever in outpatient Tanzanian children. *N Engl J Med*. Feb 27 2014;370(9):809-17. doi:10.1056/NEJMoa1214482
7. Mtove G, Hendriksen IC, Amos B, et al. Treatment guided by rapid diagnostic tests for malaria in Tanzanian children: safety and alternative bacterial diagnoses. *Malar J*. Oct 06 2011;10:290. doi:10.1186/1475-2875-10-290
8. Brent AJ, Ahmed I, Ndiritu M, et al. Incidence of clinically significant bacteraemia in children who present to hospital in Kenya: community-based observational study. *Lancet*. Feb 11 2006;367(9509):482-8. doi:10.1016/S0140-6736(06)68180-4
9. *Revised WHO Classification and Treatment of Pneumonia in Children at Health Facilities: Evidence Summaries*. 2014. *WHO Guidelines Approved by the Guidelines Review Committee*.
10. Hammitt LL, Kazungu S, Morpeth SC, et al. A preliminary study of pneumonia etiology among hospitalized children in Kenya. *Clin Infect Dis*. Apr 2012;54 Suppl 2:S190-9. doi:10.1093/cid/cir1071
11. McCollum ED, King C, Deula R, et al. Pulse oximetry for children with pneumonia treated as outpatients in rural Malawi. *Bull World Health Organ*. Dec 2016;94(12):893-902. doi:10.2471/BLT.16.173401
12. Organisation WH. WHO and UNICEF estimates of national immunization coverage Accessed 18/2/2021, 2021. https://www.who.int/immunization/monitoring_surveillance/data/mwi.pdf.
13. Harris PA, Taylor R, Thielke R, Payne J, Gonzalez N, Conde JG. Research electronic data capture (REDCap)--a metadata-driven methodology and workflow process for providing translational research informatics support. *Journal of biomedical informatics*. Apr 2009;42(2):377-81. doi:10.1016/j.jbi.2008.08.010

For peer review only - http://bmjopen.bmj.com/site/about/guidelines.xhtml

14. Fitzwater SP, Chandran A, Santosham M, Johnson HL. The worldwide impact of the seven-valent pneumococcal conjugate vaccine. *Pediatr Infect Dis J*. May 2012;31(5):501-8. doi:10.1097/INF.0b013e31824de9f6
15. Theodoratou E, Johnson S, Jhass A, et al. The effect of Haemophilus influenzae type b and pneumococcal conjugate vaccines on childhood pneumonia incidence, severe morbidity and mortality. *Int J Epidemiol*. Apr 2010;39 Suppl 1:i172-85. doi:10.1093/ije/dyq033
16. Ginsburg AS, Mvalo T, Nkwopara E, et al. Placebo vs Amoxicillin for Nonsevere Fast-Breathing Pneumonia in Malawian Children Aged 2 to 59 Months: A Double-blind, Randomized Clinical Noninferiority Trial. *JAMA Pediatr*. Nov 12 2018;doi:10.1001/jamapediatrics.2018.3407
17. Pneumonia Etiology Research for Child Health Study G. Causes of severe pneumonia requiring hospital admission in children without HIV infection from Africa and Asia: the PERCH multi-country case-control study. *Lancet*. Jun 27 2019;doi:10.1016/S0140-6736(19)30721-4
18. Gilani Z, Kwong YD, Levine OS, et al. A literature review and survey of childhood pneumonia etiology studies: 2000-2010. *Clin Infect Dis*. Apr 2012;54 Suppl 2:S102-8. doi:10.1093/cid/cir1053
19. Agweyu A, Lilford RJ, English M, Clinical Information Network Author G. Appropriateness of clinical severity classification of new WHO childhood pneumonia guidance: a multi-hospital, retrospective, cohort study. *The Lancet Global health*. Jan 2018;6(1):e74-e83. doi:10.1016/S2214-109X(17)30448-5
20. Harris M, Clark J, Coote N, et al. British Thoracic Society guidelines for the management of community acquired pneumonia in children: update 2011. *Thorax*. Oct 2011;66 Suppl 2:ii1-23. doi:10.1136/thoraxjnl-2011-200598
21. Jain S, Self WH, Wunderink RG, et al. Community-Acquired Pneumonia Requiring Hospitalization among U.S. Adults. *N Engl J Med*. Jul 30 2015;373(5):415-27. doi:10.1056/NEJMoa1500245
22. Costa LF, Queiroz DA, Lopes da Silveira H, et al. Human rhinovirus and disease severity in children. *Pediatrics*. Feb 2014;133(2):e312-21. doi:10.1542/peds.2013-2216

Figure Legends

Figure 1: Participant Flow Diagram

For peer review only

For peer review only - http://bmjopen.bmj.com/site/about/guidelines.xhtml

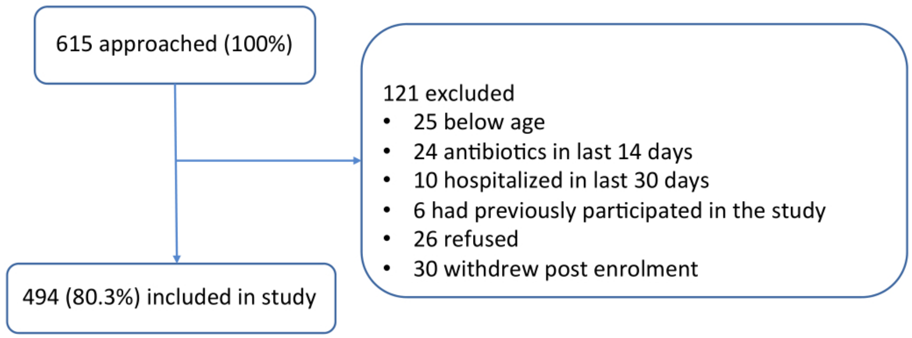

Figure 1: Participant Flow Diagram

402x165mm (72 x 72 DPI)

Supplemental file

Aetiology and Severity of Childhood Pneumonia in Primary Care in Malawi: A Cohort Study

For peer review only

Table of contents

S1: Study Location and Background health data **3**

S2: Microbiology **4**
S2a Microbiology techniques 4
Table S1 Characteristics of individual children with bacterial pneumonia 5
Table S2 Microbiology Results By month 5
Table S3 Microbiology Results By age 7

S3: Markers of severity **8**
Figure S1 Difficulty DRAWING score 8

S4: List of BIOTOPE team & Primary Affiliation **9**

For peer review only

S1: Study Location and Background health data

Mzuzu is one of the four cities in Malawi, with a population of 221,272 (National Statistics Office, 2018). Mzuzu health centre also known as Mapale or Mzimba North Health centre is stituated at the City centre of Mzuzu city while Mzuzu Central Hospital is situated at around 4 km Northwards of the city. The health centre currently serves almost the whole city as a primary health care point while the central hospital is regional referral tertiary hospital serving six districts in the northern region, over hundred health centres and serving a population of over 1.9 million people. It also serves as a secondary healthcare facility for the local (Mzuzu city) population. The majority (98.0%) of our participants were from Mapale health centre. The Central hospital initially served as a primary healthcare facility also but prior to commencement of the study the local health authority decided to maintain it as a secondary care facility only. Therefore it introduced a "bypass fee" where patients who attended the hospital clinics without being referred from a health centre/district hospital were charged MK 1,500 (approxmately 2 Euros). This led to Mapale becoming the main site of recruitment due to the focus on recruiting patients from primary care and before they had received any treatment.

UNICEF data from 2013 reports an under five mortality rate of 68 per 1,000 live births and under one mortality rate of 44 per 1,000 live births in Malawi. In 2016 the estimated coverage was 83% for pneumococcal conjugate vaccine and 84% for Haemophilus influenza type B vaccine in Malawi,[11]

HIV prevalence in antenatal clinics in the Mzimba district (where Mzuzu is located) among those who accepted testing was 9.1%[1]. A study in 2011 in Malawi showed that of 5,068 samples from infants <3 months of age , 764 were ELISA positive indicating 15.1% (14.1–16.1%) of mothers were HIV-infected and passed antibodies to their infant. Sixty-five of the ELISA-positive samples tested positive by DNA PCR, indicating a vertical transmission rate of 8.5% (6.6–10.7%). Survey data indicates 64.8% of HIV-infected mothers and 46.9% of HIV-exposed infants received some form of antiretroviral prophylaxis[2].

For peer review only - http://bmjopen.bmj.com/site/about/guidelines.xhtml

S2: Microbiology

S2a Microbiology techniques

Blood cultures

The blood culture samples were inoculated into the Bactec Peds Plus/F Culture vial (Becton Dickinson Diagnostic Instrument Systems). These bottles were incubated in an automated blood culture Bactec™ 9050 which was set at protocol limits of maximum of 7 days to declare the sample negative if no growth has been detected. All positive blood cultures were further subcultured on to agar plates and identified using traditional phenotypic methods (Blood agar, MacConkey and Chocolate agar plate) and Vitek 2 microbial detection system (Biomerieux)

Bacterial PCR

The Masterpure™ complete DNA and RNA purification kit as well as QIA Symphony Automated DNA extraction system [manufacturer. Country] which is an automated method was used to extract DNA and RNA. The extracted DNA was run on AB7500 instruments as per previously detailed methods [4,5]

Viral RT-PCR

Viral RT-PCR was performed on nasopharyngeal and oropharyngeal swabs using a Luminex® MAGPIX® instrument [manufacturer, country] with xPONENT® and SYNCT™ software ,in line with manufacturer's instructions. The panel used was NxTAG respiratory pathogen. The pathogens targeted in this assay were; Influenza A, Influenza A H1, Influenza B, Influenza A H3, Respiratory Syncytial Virus A, Respiratory Syncytial Virus B, Coronavirus 229E, Coronavirus OC43, Coronavirus NL63, Coronavirus HKU1, Human Metapneumovirus, Rhinovirus/Enterovirus Adenovirus, Parainfluenza 1, Parainfluenza 2, Parainfluenza 3, Parainfluenza 4, Human Bocavirus, *Chlamydophila pneumoniae, Mycoplasma pneumoniae, Legionella pneumophila*

Table S1 Characteristics of individual children with bacterial pneumonia

Bacteria	Method of detection	Age (years)	Gender	Malaria	HIV	Antibiotic
S. pneumoniae & Haemophilus spp.	Blood PCR	1.1	M	Neg	Neg	Cotrimoxazole
S. pneumoniae	Blood PCR	2.3	M	Pos	Neg	Cotrimoxazole
S. pneumoniae & Haemophilus spp.	Blood PCR	1.1	M	Pos	Neg	Cotrimoxazole
S. pneumoniae & Haemophilus spp.	Blood PCR	1.7	M	Neg	Neg	Unknown
S aureus	Blood culture	2.4	M	Neg	Neg	Cotrimoxazole
S aureus	Blood culture	1.7	M	Neg	Neg	Cotrimoxazole
Haemophilus spp.	Blood PCR	2.8	F	Neg	Neg	Unknown
Haemophilus spp.	Blood PCR	2.7	M	Neg	Neg	Amoxicillin
C. pneumoniae	OP/NP swab PCR	3.6	M	Neg	Neg	Amoxicillin
M. pneumoniae	OP/NP swab PCR	2.6	F	Neg	Pos	Amoxicillin
M. pneumoniae	OP/NP swab PCR	1.7	M	Neg	Neg	Amoxicillin
M. pneumoniae	OP/NP swab PCR	2.4	F	Neg	Neg	Unknown
C. pneumoniae	OP/NP swab PCR	0.6	M	Neg	Unk	Amoxicillin

Table S2 Microbiology Results By month

Variable	Statistic	Mar	Apr	May	Jun
**Bacterial**					
S. pneumoniae	n (%)	0/12 (0%)	0/40 (0%)	3/185 (1.6%)	1/257 (0.4%)
S. auerus	n (%)	0/12 (0%)	0/40 (0%)	0/185 (0%)	2/257 (0.4%)
Haemophilus spp.	n (%)	0/12 (0%)	0/40 (0%)	2/185 (1.1%)	3/257 (1.2%)

For peer review only

M. pneumoniae	n (%)	0/12 (0%)	1/40 (2.5%)	2/185 (1.1%)	0/257 (0%)
C._pneumoniae	n (%)	0/12 (0%)	0/40 (0%)	1/185 (0.5%)	1/257 (0.4%)
Other_culture	n (%)	0/12 (0%)	0/40 (0%)	1/185 (0.5%)	2/257 (0.8%)
**Viral[1]**					
Adenovirus	n (%)	6/12 (50%)	10/40 (25%)	33/185 (17.8%)	30/257 (11.7%)
Bocavirus	n (%)	2/12 (16.7%)	7/40 (17.5%)	37/185 (20%)	47/257 (18.3%)
CoV* HKU	n (%)	0/12 (0%)	0/40 (0%)	0/185 (0%)	0/257 (0%)
CoV* NL63	n (%)	0/12 (0%)	1/40 (2.5%)	0/185 (0%)	0/257 (0%)
CoV* 229E	n (%)	0/12 (0%)	0/40 (0%)	0/185 (0%)	1/257 (0.4%)
CoV* OC43	n (%)	0/12 (0%)	0/40 (0%)	0/185 (0%)	1/257 (0.4%)
Influenza A	n (%)	0/12 (0%)	10/40 (25%)	42/185 (22.7%)	23/257 (8.9%)
Influenza AH1	n (%)	0/12 (0%)	10/40 (25%)	37/185 (20%)	16/253 (6.2%)
Influenza AH3	n (%)	0/12 (0%)	0/40 (0%)	3/185 (1.6%)	3/257 (1.2%)
Influenza B	n (%)	0/12 (0%)	0/40 (0%)	2/185 (1%)	6/257 (2.3%)
HMPV+	n (%)	2/12 (16.7%)	10/40 (25%)	33/185 (17.8%)	27/257 (10.5%)
Parainfluenza 1	n (%)	0/12 (0%)	2/40 (5%)	5/182 (2.7%)	4/257 (1.5%)
Parainfluenza 2	n (%)	0/12 (0%)	3/40 (7.5%)	4/185 (2.2%)	3/257 (1.2%)
Parainfluenza 3	n (%)	1/12 (8.3%)	0/40 (0%)	3/185 (1.6%)	0/257 (0%)
Parainfluenza 4	n (%)	1/12 (8.3%)	3/40 (7.5%)	7/185 (3.8%)	5/257 (1.9%)
RSV// A	n (%)	0/12 (0%)	1/40 (2.5%)	22/185 (11.9%)	129/257 (50.2%)
RSV// B	n (%)	0/12 (0%)	2/40 (5%)	12/185 (6.5%)	35/257 (13.6%)
Rhino/enterovirus	n (%)	10/12 (83.3%)	22/40 (55%)	132/185 (71.3%)	128/257 (49.8%)

* = Coronavirus

+ = Human metapneumovirus

// = Respiratory syncytial virus

Table S3 Microbiology Results By age

Variable	Statistic	2-11 months	12-35 months	36+ months
**Bacterial**				
S. pneumoniae	n (%)	2/157 (1.3%)	2/252 (0.8%)	0/85 (0%)
S. auerus	n (%)	0/157 (0%)	2/252 (0.8%)	0/85 (0%)
Haemophilus spp.	n (%)	2/157 (1.4%)	3/252 (1.2%)	0/85 (0%)
M. pneumoniae	n (%)	0/157 (0%)	3/252 (1.2%)	0/85 (0%)
C. pneumoniae	n (%)	0/157 (0%)	1/252 (0.4%)	1/85 (1.2%)
**Viral**				
Adenovirus	n (%)	12/157 (7.6%)	48/252 (19%)	17/85 (20%)
Bocavirus	n (%)	25/157 (15.9%)	52/252 (20.6%)	14/85 (16.5%)
CoV* HKU	n (%)	0/157 (0%)	0/252 (0%)	0/85 (0%)
CoV* NL63	n (%)	0/157 (0%)	1/252 (0.4%)	0/85 (0%)
CoV* 229E	n (%)	0/157 (0%)	1/252 (0.4%)	0/85 (0%)
CoV* OC43	n (%)	0/157 (0%)	1/252 (0.4%)	0/85 (0%)
Influenza A	n (%)	8/157 (5%)	40/252 (15.9%)	26/85 (30.6%)
Influenza AH1	n (%)	8/157 (5%)	30/252 (11.9%)	24/85 (28.2%)
Influenza AH3	n (%)	0/157 (0%)	5/252 (1.9%)	1/85 (1.2%)
Influenza B	n (%)	2/157 (1.3%)	3/252 (1.2%)	2/84 (2.3%)
HMPV+	n (%)	19/157 (12.1%)	41/252 (16.3%)	11/84 (12.9%)
Parainfluenza 1	n (%)	2/157 (1.3%)	4/252 (1.6%)	5/85 (5.8%)
Parainfluenza 2	n (%)	2/157 (1.3%)	5/252 (2%)	2/85 (2.3%)
Parainfluenza 3	n (%)	1/157 (0.6%)	3/252 (1.2%)	0/85 (0%)
Parainfluenza 4	n (%)	4/157 (2.5%)	9/252 (3.6%)	3/85 (3.5%)
RSV// A	n (%)	61/157 (38.8%)	76/252 (30.2%)	10/85 (11.8%)
RSV// B	n (%)	18/157 (11.4%)	22/252 (8.7%)	7/85 (8.2%)
Rhino/enterovirus	n (%)	83/157 (52.9%)	148/252 (58.7%)	53/85 (62.3%)

* = Coronavirus

+ = Human metapneumovirus

// = Respiratory syncytial virus

For peer review only

S3: Markers of severity

Figure S1 Difficulty DRAWING score

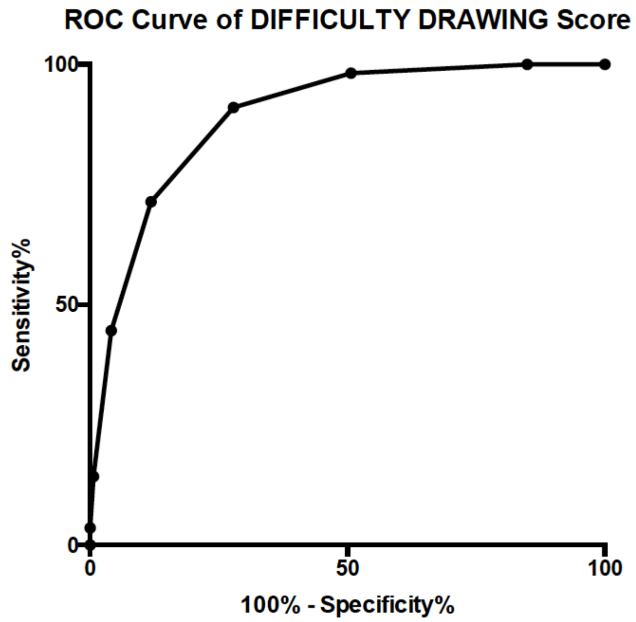

DIFFICULTY DRAWING breath score = 1 for each of:

DIFFICULTY breathing
Deep breathing
Respiratory Rate > 70 bpm
Age <2 years
Wheeze
INdrawing lower chest wall
Grunting

Area under the ROC curve
Area 0.8918
Std. Error 0.02002
95% confidence interval 0.8525 to 0.9310
P value< 0.0001
Data
Controls (Not Hospitalised) 438
Patients (Hospitalised) 56
Missing Controls0
Missing Patients0

S4: List of BIOTOPE team & Primary Affiliation

gHealth Research Group, School of Medicine, UCD
Joe Gallagher
Nadezhda Glezeva
Mark Ledwidge (statistics)
John O Donoghue
Dermot Wildes

Irish Meningitis and Sepsis Reference Laboratory
Richard Drew
Desiree Bennett
Nicola O'Sullivan

Luke International
Joseph Wu
Hsin yi Lee

Mzuzu Central Hospital/Mapale Health Centre
Master Chisale
Maynard Khondowe,
Themba Luwe
Hastings Scott
Chikondi Chimbatata
Cecilia Mwalilino
Kelvin Ulili
Lameck Manda
Joseph Phiri
Alinafe Mpicha
Isaac Namwiri

National Virus Reference Laboratory, Ireland
Cillian De Gascun
Suzie Coughlan
Linda Dunford
Christina Byrne

Queens University Belfast
Chris Watson

Royal College of Surgeons in Ireland
Sudip Das

Eoin O Connell (statistics)

For peer review only

1. Manda S, Masenyetse L, Cai B, Meyer R. Mapping HIV prevalence using population and antenatal sentinel-based HIV surveys: a multi-stage approach. Popul Health Metr 2015;13:22.
2. Sinunu MA, Schouten EJ, Wadonda-Kabondo N, et al. Evaluating the impact of prevention of mother-to-child transmission of HIV in Malawi through immunization clinic-based surveillance. PloS one 2014;9:e100741.
3. International. NSONMaI. Malawi Demographic and Health Survey 2015-16: Key Indicators Report. 2016.
4. Meyler KL, Meehan M, Bennett D, Cunney R, Cafferkey M. Development of a diagnostic real-time polymerase chain reaction assay for the detection of invasive Haemophilus influenzae in clinical samples. Diagn Microbiol Infect Dis. 2012 Dec;74(4):356-62. doi: 10.1016/j.diagmicrobio.2012.08.018. Epub 2012 Sep 25. PMID: 23017260.
5. [1]

Murphy J, O' Rourke S, Corcoran M, O' Sullivan N, Cunney R, Drew R. Evaluation of the Clinical Utility of a Real-time PCR Assay for the Diagnosis of Streptococcus pneumoniae Bacteremia in Children: A Retrospective Diagnostic Accuracy Study. Pediatr Infect Dis J. 2018 Feb;37(2):153-156. doi: 10.1097/INF.0000000000001772. PMID: 29076932.

review only

STROBE Statement—checklist of items that should be included in reports of observational studies

	Item No.	Recommendation	Page No.
**Title and abstract**	1	(*a*) Indicate the study's design with a commonly used term in the title or the abstract	1
		(*b*) Provide in the abstract an informative and balanced summary of what was done and what was found	3
**Introduction**			
Background/rationale	2	Explain the scientific background and rationale for the investigation being reported	5
	3	State specific objectives, including any prespecified hypotheses	6
**Methods**			
Study design	4	Present key elements of study design early in the paper	6
Setting	5	Describe the setting, locations, and relevant dates, including periods of recruitment, exposure, follow-up, and data collection	6
Participants	6	(*a*) *Cohort study*—Give the eligibility criteria, and the sources and methods of selection of participants. Describe methods of follow-up	6-7
Variables	7	Clearly define all outcomes, exposures, predictors, potential confounders, and effect modifiers. Give diagnostic criteria, if applicable	8
Data sources/ measurement	8*	For each variable of interest, give sources of data and details of methods of assessment (measurement). Describe comparability of assessment methods if there is more than one group	*8*
Bias	9	Describe any efforts to address potential sources of bias	
Study size	10	Explain how the study size was arrived at	9

Continued on next page

For peer review only

Quantitative variables	11	Explain how quantitative variables were handled in the analyses. If applicable, describe which groupings were chosen and why	
Statistical methods	12	(*a*) Describe all statistical methods, including those used to control for confounding	9
		(*b*) Describe any methods used to examine subgroups and interactions	
		(*c*) Explain how missing data were addressed	9
		(*d*) *Cohort study*—If applicable, explain how loss to follow-up was addressed	9
		(*e*) Describe any sensitivity analyses	NA
**Results**			
Participants	13*	(a) Report numbers of individuals at each stage of study—eg numbers potentially eligible, examined for eligibility, confirmed eligible, included in the study, completing follow-up, and analysed	Figure 1
		(b) Give reasons for non-participation at each stage	Figure 1
		(c) Consider use of a flow diagram	Figure 1
Descriptive data	14*	(a) Give characteristics of study participants (eg demographic, clinical, social) and information on exposures and potential confounders	Table 1
		(b) Indicate number of participants with missing data for each variable of interest	Table 1
		Summarise follow-up time (eg, average and total amount)	8
Outcome data	15*	Report numbers of outcome events or summary measures over time	*Table 1*
Main results	16	(*a*) Give unadjusted estimates and, if applicable, confounder-adjusted estimates and their precision (eg, 95% confidence interval). Make clear which confounders were adjusted for and why they were included	10-12
		(*b*) Report category boundaries when continuous variables were categorized	
		(*c*) If relevant, consider translating estimates of relative risk into absolute risk for a meaningful time period	Not applicable

Continued on next page

For peer review only

Other analyses	17	Report other analyses done—eg analyses of subgroups and interactions, and sensitivity analyses	
**Discussion**			
Key results	18	Summarise key results with reference to study objectives	12
Limitations	19	Discuss limitations of the study, taking into account sources of potential bias or imprecision. Discuss both direction and magnitude of any potential bias	14
Interpretation	20	Give a cautious overall interpretation of results considering objectives, limitations, multiplicity of analyses, results from similar studies, and other relevant evidence	14-15
Generalisability	21	Discuss the generalisability (external validity) of the study results	14
**Other information**			
Funding	22	Give the source of funding and the role of the funders for the present study and, if applicable, for the original study on which the present article is based	23

*Give information separately for cases and controls in case-control studies and, if applicable, for exposed and unexposed groups in cohort and cross-sectional studies.

Note: An Explanation and Elaboration article discusses each checklist item and gives methodological background and published examples of transparent reporting. The STROBE checklist is best used in conjunction with this article (freely available on the Web sites of PLoS Medicine at http://www.plosmedicine.org/, Annals of Internal Medicine at http://www.annals.org/, and Epidemiology at http://www.epidem.com/). Information on the STROBE Initiative is available at www.strobe-statement.org.

For peer review only

For peer review only - http://bmjopen.bmj.com/site/about/guidelines.xhtml

BMJ Open

Aetiology and Severity of Childhood Pneumonia in Primary Care in Malawi: A Cohort Study

Journal:	*BMJ Open*
Manuscript ID	bmjopen-2020-046633.R2
Article Type:	Original research
Date Submitted by the Author:	29-Jun-2021
Complete List of Authors:	Gallagher, Joe; University College Dublin College of Health Sciences, gHealth Research Institute<br>Chisale, Master; Faculty of Science Technology and Innovations, Mzuzu University, Malawi, Biological Science Dept<br>Das, Sudipto; Royal College of Surgeons in Ireland<br>Drew, Richard; Irish Meningitis and Sepsis Reference Laboratory<br>Gleseva, Nadezhda; University College Dublin College of Health Sciences, gHealth Research Institute<br>Wildes, Dermot; University College Dublin College of Health Sciences, gHealth Research Institute<br>De Gascun, Cillian; National Virus Reference Laboratory<br>Wu, Tsung-Shu; Luke International Norway; Overseas Mission Department, Pingtung Christian Hospital<br>Ledwidge, Mark; University College Dublin College of Health Sciences, gHealth Research Institute<br>Watson, Chris; Queen's University Belfast Wellcome-Wolfson Institute for Experimental Medicine
**Primary Subject Heading**:	Paediatrics
Secondary Subject Heading:	Global health, Evidence based practice, Public health
Keywords:	Epidemiology < TROPICAL MEDICINE, Microbiology < PATHOLOGY, Paediatric infectious disease & immunisation < PAEDIATRICS, Paediatric thoracic medicine < PAEDIATRICS, PRIMARY CARE

SCHOLARONE™
Manuscripts

I, the Submitting Author has the right to grant and does grant on behalf of all authors of the Work (as defined in the below author licence), an exclusive licence and/or a non-exclusive licence for contributions from authors who are: i) UK Crown employees; ii) where BMJ has agreed a CC-BY licence shall apply, and/or iii) in accordance with the terms applicable for US Federal Government officers or employees acting as part of their official duties; on a worldwide, perpetual, irrevocable, royalty-free basis to BMJ Publishing Group Ltd ("BMJ") its licensees and where the relevant Journal is co-owned by BMJ to the co-owners of the Journal, to publish the Work in this journal and any other BMJ products and to exploit all rights, as set out in our licence.

The Submitting Author accepts and understands that any supply made under these terms is made by BMJ to the Submitting Author unless you are acting as an employee on behalf of your employer or a postgraduate student of an affiliated institution which is paying any applicable article publishing charge ("APC") for Open Access articles. Where the Submitting Author wishes to make the Work available on an Open Access basis (and intends to pay the relevant APC), the terms of reuse of such Open Access shall be governed by a Creative Commons licence – details of these licences and which Creative Commons licence will apply to this Work are set out in our licence referred to above.

Other than as permitted in any relevant BMJ Author's Self Archiving Policies, I confirm this Work has not been accepted for publication elsewhere, is not being considered for publication elsewhere and does not duplicate material already published. I confirm all authors consent to publication of this Work and authorise the granting of this licence.

For peer review only

Aetiology and Severity of Childhood Pneumonia in Primary Care in Malawi: A Cohort Study

Joe Gallagher, MD[1*], Master Chisale, MSc[1,2], Sudipto Das, PhD[3], Richard Drew, MD[3,4], Nadezhda Gleseva, PhD[1], Dermot M Wildes[1], MB, Cillian De Gascun, MD[5], Tsung-Shu Joseph Wu, MSc[6,7], Mark Ledwidge, PhD[1], Chris J Watson, PhD[1,8].

On behalf of the BIOTOPE team

[1]gHealth Research Group, UCD Conway Institute, School of Medicine, University College Dublin, Ireland.

[2]Biological Science Department, Faculty of Science, Technology & Innovations, Mzuzu University, Malawi

[3]Royal College of Surgeons in Ireland, Dublin, Ireland.

[4] Irish Meningitis and Sepsis Reference Laboratory, Temple Street, Dublin 2.

[5] UCD National Virus Reference Laboratory, University College Dublin, Ireland.

[6] Luke International Norway, Mzuzu, Malawi.

[7]Overseas Mission Department, Pingtung Christian Hospital, Taiwan.

[8] Wellcome-Wolfson Institute for Experimental Medicine, Queen's University Belfast, Northern Ireland.

For peer review only - http://bmjopen.bmj.com/site/about/guidelines.xhtml

* Corresponding author:

Dr. Joe Gallagher,

School of Medicine,

University College Dublin,

Dublin.

Tel: 053 9421446

Fax: 053 9422526

Email: joejgallagher@gmail.com

Manuscript word count: 2,358 (Excluding Abstract, Tables, Figures & References)

Ethics - The protocol and related documents were successfully vetted by the National Health Science Research Committee of Malawi - Ethics approval number 15/11/1532

For peer review only

Abstract

Objective:

To determine the aetiology of community acquired pneumonia in children presenting to primary care in Northern Malawi, and to ascertain predictors for identification of children requiring hospitalisation.

Design: The BIOmarkers TO diagnose PnEumonia (BIOTOPE) study was a prospective cohort study conducted from March to June 2016.

Setting: Primary care in Northern Malawi.

Patients: 494 children aged 2 months to 59 months with World Health Organization defined pneumonia.

Main Outcome(s) and Measure(s): Number of children with bacterial infection identified and the sensitivity/specificity of WHO markers of severity for need for hospitalisation.

Results:

13 (2.6%) children had a bacterium consistent with pneumonia identified. A virus consistent with pneumonia was identified in in 448 (90.7%) of children. 56 children were admitted to hospital and two children died within 30 days. 442 (89.5%) received antibiotic therapy. Eleven children (2.6%) had HIV. WHO severity markers at baseline demonstrated poor sensitivity for the need for hospitalization with a sensitivity of 0.303 (95% confidence interval 0.188-0.441) and a specificity 0.9 (95% confidence

For peer review only

interval 0.868 to 0.926). A prediction rule to indicate the need for hospitalization was developed.

Conclusions and Relevance:

The low rate of bacterial infection and high use of antibiotics in the setting of high immunisation rates highlights the changing profile of childhood pneumonia. Similarly, the markers of need for hospitalization may have changed in the setting of extended immunisation. Further studies are required to examine this.

Article Summary:

- The pneumococcal vaccine was introduced in 2012 in Malawi and this study provides data on the aetiology and severity markers in primary care following the introduction of this vaccine.
- It demonstrates the significant reduction in bacterial infection compared to the previous studies undertaken using similar methods prior to introduction of this vaccine in Africa
- It also highlights that the severity markers in childhood pneumonia are changing and this is important since these markers are still used in primary care in Africa for the determination of referral to hospital.
- The study was in an urban area with high immunisation rates over one season and the lack of a control group (i.e., well children) limited our ability to verify the clinical significance of most viral pathogens and atypical bacteria

For peer review only

INTRODUCTION

Despite recent advances in reducing childhood mortality, pneumonia remains a major cause of morbidity and mortality among children aged under five years, with an estimated 935,000 deaths per annum.(1) Allied to this is the growing concern regarding antimicrobial resistance driven by overuse of antibiotics and intercontinental spread of this resistance.(2-4) The increased use of universal immunisation, including haemophilus influenzae type B (Hib) and pneumococcal vaccines are likely to have altered the proportion with viral illness. This may also have altered the aetiology, severity, and host response in childhood pneumonia. A greater understanding and characterisation of these factors in primary care is required to continue advances in reducing childhood mortality from pneumonia.

Current strategies for the management of childhood pneumonia in developing countries focus on the identification of the illness and provision of antibiotics, despite the low reported prevalence of bacterial infection.(5) Most studies have focused on the hospitalised population, and yet in developing countries, it is in the community where these patients are most likely to present. Therefore, contemporary estimates of the microbiological causes of pneumonia and predictors of hospitalization would be of benefit. The use of microbiological techniques is hampered by the high rate of carriage of bacteria and viruses in children.(6) Finally, WHO markers of severity, which are used to guide referral for hospitalization in primary care, are mostly based on clinical symptoms alone and advise the use of pulse oximetry to help determine severity if available. However oximeters are rarely available in primary care in countries such as Malawi.(7)

review only

The BIOTOPE study (BIOmarkers TO diagnose PnEumonia) was a prospective cohort study and this paper reports on the aetiology of community acquired pneumonia in children presenting in primary care in Northern Malawi and predictors of children requiring hospitalization.

METHODS

For peer review only

Study Sites & Population

From March to June 2016, we recruited children at Mapale Health Centre and the outpatient department of Mzuzu Central Hospital in Mzuzu, Northern Malawi – both of which serve as primary care facilities for this urban area. Shortly after study commencement, the outpatient department of Mzuzu Central Hospital ceased to provide primary care services. As such, 98% of participants were recruited from Mapale Health Centre. Mapale Health Centre currently serves the whole city as a primary health care facility (population of approximately 220,000) The under-five population in this setting is characterised by a high burden of infectious disease including acute respiratory infections (ARI), diarrhoea, and malaria. In 2016 the estimated coverage was 83% for pneumococcal conjugate vaccine and 84% for Haemophilus influenza type B vaccine in Malawi.(8)

(Further details on site are available in the supplemental file)

For peer review only - http://bmjopen.bmj.com/site/about/guidelines.xhtml

Study participants:

Children presenting to the primary care facilities were assessed for study enrolment. Children aged 2-59 months were deemed eligible to participate in the study if the main presenting complaint was cough or difficulty breathing associated with tachypnoea (defined as >50 breaths per minute if aged 2-11 months or > 40 breaths per minute age if aged 12-59 months) or chest in-drawing. This is the current WHO clinical case definition of pneumonia.(5) Exclusion criteria listed were as follows: those discharged from hospital in the preceding 30 days; those who had completed a course of antibiotics within 14 days of presentation; or those who had received antibiotics prior to clinical assessment for this illness. The study details were discussed with a parent/guardian of any patient who fit our inclusion criteria, who in turn provided informed consent in a written format. The protocol and related documents were approved by the National Health Science Research Committee of Malawi and the Mzuzu Central Hospital Research Committee. Patients received timely treatment and supportive care as required. The study was conducted in compliance with the ethical standards as outlined by two ethics committees and was aligned with the values outlined by the Declaration of Helsinki. (National Health Research Committee of Malawi Ethics Approval #15/11/1532.

Study Procedures

Clinical Assessment

In order to record clinical data, a standardized report form was provided to our investigators incorporating demographics, risk factors, and items relevant to a pneumonia presentation. Immunisation records are held by patients in a patient held

For peer review only - http://bmjopen.bmj.com/site/about/guidelines.xhtml

record known as the "health passport". Only those who had a health passport with recorded immunisations were used for purpose of recording immunisation status. Where possible, children were followed up by contacting a caregiver at 7 days and 30 days following initial assessment by telephone. All children had a full blood count and malaria rapid diagnostic test. HIV rapid diagnostic testing was offered to all children aged over 12 months and HIV PCR testing to all children aged 2 months to 11 months.

Microbiologic and Other Investigations

For all patients, blood samples (8 ml) and pooled naso/oro-pharyngeal swabs were collected for microbiological investigation on site, and for further serologic and molecular analysis in Ireland. A systematic set of investigations were performed for all patients. Blood cultures were processed locally. Blood samples for PCR for *Streptococcus pnuemoniae*, *Staphylococcus aureus* and *Haemophilus influenzae* along with naso/oro-pharyngeal swabs for respiratory viruses and atypical bacteria were processed in Ireland. (Further details in the supplemental file)

Definitive Diagnosis Based on Predefined Criteria

The final diagnosis (in some cases, diagnoses) was ascertained for each patient using pre-determined criteria for both clinical and microbiological assessment. A case was deemed to be "severe" should the child's condition fulfil WHO criteria for this state. Bacterial pneumonia was diagnosed on a positive blood culture or positive blood PCR for a relevant organism or positive nasopharyngeal swab for C. pneumoniae or M.

For peer review only - http://bmjopen.bmj.com/site/about/guidelines.xhtml

pneumonia. Laboratory data were combined with the predetermined clinical criteria to establish each diagnosis.

Statistical Analyses

The overall aim of this project was to determine blood and urine biomarkers of host response in bacterial pneumonia in a cohort of children age 2- 59 months in a single centre in Malawi. Based on the study by D'Acrement et al it was estimated that the prevalence of bacterial infection among febrile children with pneumonia would be 20%.(9) The required sample size for our study based on this prevalence was given by the formula n= 4pq/d2 where p = prevalence q = 100-p and d= the precision of the estimate. Assuming a relative precision of 20% and based on previous pilot work on follow up and sample analysis this resulted in a required sample size of n= 490.

Data were entered into REDCap electronic data capture tools.(10) All statistical analyses were performed using R software (R Foundation for Statistical Computing). Sensitivity and specificity of WHO severity markers for hospitalization were calculated, with the Clopper-Pearson method used for their confidence intervals. Logistic regression using pre-defined variables was undertaken to identify a new clinical prediction model and the Area under the curve (AUC) was calculated by area summation, Tjur r-squared estimate and Hanley & McNeil's (1982) SE formula was used for AUC confidence intervals. A simplified clinical prediction rule was developed by selecting independent predictors of hospitalization measurable from multivariable analyses, allocating a single point for each characteristic and creating a

For peer review only - http://bmjopen.bmj.com/site/about/guidelines.xhtml

sum score for each patient. We applied Receiver Operated Characteristic (ROC) analysis at various point thresholds of the score.

Patient & Public Involvement Statement

Patients were not involved in concept design, participant recruitment or study conduct at any stage from inception to completion of this project.

For peer review only

RESULTS

Study Sample

A total of 615 children were approached for inclusion in this study. (Figure 1). Demographic and clinical characteristics of the children included in the study are described in Table 1. At day seven, 225 caregivers were contactable by phone and 100% of children were alive. At day thirty, 195 caregivers were contactable and two children had died.

Immunisations

317 patients had a patient passport with immunisation record available. 239 of these patients were over the age of 12 months, and 109 were over 24 months of age. 98 (89.9%) of those aged 24 months or over who had immunisation records available had received full immunisations. Furthermore, of the 56 patients who were hospitalised, 45 had immunization records available. We age and sex matched this group in a 1:2 ratio with 90 patients who were not-hospitalised and had

For peer review only - http://bmjopen.bmj.com/site/about/guidelines.xhtml

immunization records available. The median number of immunisations amongst the hospitalized cohort was significantly lower than the non-hospitalised cohort (9 [IQR 7,10] vs. 10 [IQR10,10], p=0.003). Children without full complement of vaccinations required by age one year were more likely to be hospitalised (RR 5.5, [95% CI: 1.88-16.1], p=0.008) than those with the correct vaccinations in age and sex adjusted analyses.

Microbiological Analyses

A bacterium consistent with pneumonia was identified in 13 (2.6%) children. In three cases, more than one bacterial cause was identified in the same child. Respiratory viruses were found in 12 of the 13 children considered to have bacterial pneumonia. In 448 (90.7%) of children a respiratory virus alone was identified on testing. Full details of the microbiological analyses are provided in Table 2 and the supplemental file. In 33 (6.7%) cases, no organism associated with pneumonia was identified.

WHO Severity Criteria and Hospitalization

WHO severity signs and other variables were recorded prospectively. The sensitivity and specificity of these are in Table 3.

Using logistic regression, further models were developed with significant improvement in discriminant value. In multivariable modeling, the following seven variables were predictive of hospitalization: Difficulty breathing, Deep breathing,

For peer review only - http://bmjopen.bmj.com/site/about/guidelines.xhtml

Respiratory rate, Age, Wheeze, Lower chest wall indrawing. The AUC of the fully adjusted model for prediction of hospitalization was equal to 0.92 (95% CI 0.90, 0.93, $p<0.0001$). To create a simplified version of this model, we assigned a score of 1 to each sign or symptom (including categorical treatment of Age <2 years and Respiratory Rate >70 bpm) and generated a simple, 7 point "DIFFICULTY DRAWING breath" score for each patient, which predicted need for hospitalization with an AUC of 0.91 (95% CI 0.87 to 0.95, $p<0.0001$). A score of 0-1 was associated with low need for hospitalization, 2-3 was intermediate and a score of 4 or greater suggested a high need for hospitalization. Also, a score of 3 or more had a sensitivity of 87.5% (95% CI 95.9, 94.8) and a specificity of 84.0% (95% CI 80.2, 87.4) for hospitalization. (Supplemental file). This score is predictive of hospitalisation, independent of age, malaria status, HIV status, number of vaccinations, number of people usually sleeping in the same room as the child, presence of chimney for indoor fire, availability of electricity in the home, distance from the nearest health clinic and staff designation of severe pneumonia using WHO IMCI criteria. The DIFFICULTY DRAWING breath score was also independently associated with staff designation of severe pneumonia. However, we observed that there was no relationship between staff designation of severe pneumonia using WHO criteria and the decision to admit. This score does require added calculation compared to the WHO severity markers which only require any one of the items and also requires a decision threshold as to the level that requires referral to hospital. However, multicomponent scores are common in clinical practice and we believe that evaluation of such scores and consideration of trials integrated them into electronic decision supports is warranted.(11) The score here also has a high sensitivity compared to the WHO score which has a high specificity. We believe

For peer review only - http://bmjopen.bmj.com/site/about/guidelines.xhtml

that in primary care a score with a high sensitivity is more appropriate to ensure that false negatives are limited.(12) These aspects will need to be evaluated and validated in future studies.

DISCUSSION

This study demonstrates that in an area of high immunisation uptake that the prevalence of bacterial pneumonia in children presenting in primary care with clinically defined pneumonia is low. The introduction of both Haemophilus influenzae type b and pneumococcal conjugate vaccines has been shown to reduce the incidence of severe bacterial infections and hospitalization among children.(13, 14) This data is consistent with the high uptake of immunisations in our cohort. A recent study has also shown that, in Malawi, the number of children with non-severe, fast-breathing pneumonia that needed amoxicillin treatment, for one child to benefit was 33 which is supportive of our findings also.(15) The number of children with bacterial pneumonia has declined significantly since a previous study in the community in Eastern Africa performed prior to the introduction of pneumococcal vaccine although, this used different microbiological techniques to assign diagnoses.(9) The PERCH study which examined the causes of severe childhood pneumonia requiring hospital admission has also shown a predominantly viral aetiology even in this severe subset.(16) Overall, very few studies have been conducted in primary care in the era prior to the introduction of pneumococcal vaccine.(15-19) Given that most children with pneumonia will present initially to

For peer review only - http://bmjopen.bmj.com/site/about/guidelines.xhtml

primary care, there is a clear need for further evaluation of both the aetiology and severity profile of childhood pneumonia in this setting.

The markers of severity (defined as hospitalization) in the BIOTOPE study are different from that outlined in current WHO guidelines even those these are the guidelines used to guide who should be referred to hospital. Our study shows that WHO severity criteria are present in a minority (30.4%) of children hospitalised, consistent with recent work showing that 39% of fatal cases of pneumonia were defined as having non-severe pneumonia requiring only home treatment by the 2013 revision.(18) The fact that the WHO criteria would have discharged these children with oral antibiotics highlights the need for new markers of severity, as these guidelines are used to guide primary care workers in their decision-making regarding escalation of care. Our study highlights the changing pattern of markers of severity for hospitalization and generates new markers of severity that could be used in the community. We present a new 7-point clinical prediction rule (DIFFICULTY DRAWING Breath score) based on independent predictors of severity with a high predictive power for severity, defined as need for hospitalization. This will need to be evaluated in future studies.

This study involves an urban centre in an area of high immunisation and results may differ in different settings, particularly in locations with low immunisation uptake rates or where healthcare may not be easily accessed e.g. remote areas. Infection

For peer review only - http://bmjopen.bmj.com/site/about/guidelines.xhtml

rates for both respiratory viruses and malaria are subject to significant seasonal variation, as such, had the study been conducted during an alternative time period or in an alternative geographical location, the results may have differed significantly. However, given the study site was representative of Malawi and the Mzuzu region as a whole in terms of immunisation rates, we believe our findings may be generalised and applied to Malawi as a nation and (to a certain extent) other nations of sub-Saharan Africa. More studies are required in different epidemiologic settings to generate further data. The lack of a control group (i.e., well children) limited our ability to verify the clinical significance of most viral pathogens and atypical bacteria. However, all children were included based on a clinical definition of pneumonia and therefore had pre-existing illness signs. Our rate of identification of a possible pathogen was high and this likely reflects the use of molecular techniques. The lack of chest radiography may be seen as a limitation but previous studies have shown it is not predictive of aetiology and does not improve outcomes.(19) Also, plain film radiographs would not be routinely available in primary care. The use of hospitalisation as a marker of severity may be seen as a limitation as hospitalisation may vary based on hospital practice and health seeking behaviour. However this study was based in primary care and hospitalisation is a useful standard to guide those who should be referred from primary care. Larger studies to evaluate and validate scores for markers such as mortality are required

Our findings indicate that among pre-school aged children in a region with high rates of immunisation, a high percentage of WHO clinically defined pneumonia are likely to

For peer review only - http://bmjopen.bmj.com/site/about/guidelines.xhtml

be of viral origin. It is known that respiratory viruses, including rhinoviruses, can cause severe lower respiratory tract disease, particularly in infants and young children.(20,21) There is also a change in the clinical markers of severe illness, as defined by hospitalization. The current WHO markers of severity have a low sensitivity for hospitalization. This has important implications for the management of childhood pneumonia in the community and may reflect the change in clinical signs in the era of high immunisation uptake.

Conclusion:

In Mzuzu, Malawi, the vast majority of children with a WHO clinical definition of pneumonia had a viral illness and thus did not require an antibiotic. Current guidelines suggest all of these children should receive antibiotics. This subjects the community to accelerated development of antimicrobial resistance. The current definition of severe clinical signs used by the WHO has a poor sensitivity and this is potentially attributable to the high uptake of immunisations against respiratory bacteria in this cohort, with a corresponding evolution of disease presentation. Further study is warranted to determine if a revision of current WHO guidelines is required, thereby ensuring children are appropriately are referred to hospital. The

For peer review only - http://bmjopen.bmj.com/site/about/guidelines.xhtml

score presented here provides a practical alternative, should it be more widely validated.

For peer review only

For peer review only - http://bmjopen.bmj.com/site/about/guidelines.xhtml

Table 1 Baseline demographic and clinical characteristics

**Demographics**		**Total Population N=494**	**Not hospitalised (n=438)**	**Hospitalised (n=56)**	
Age (months)	Median [IQR]	18 [10-30]	19 [10-32]	13 [6-21]	<0.001
Age 2 - 11 months	n (%)	157/494 (31.8%)	132/438 (30.1%)	25/56 (44.6%)	0.033
Age 12 - 35 months	n (%)	252/494 (51.0%)	224/438 (51.1%)	28/56 (50.0%)	NS
Age 36 - 60 months	n (%)	85/494 (17.2%)	82/438 (18.7%)	3/56 (5.3%)	0.013
Male	n (%)	271/494 (54.8%)	240/438 (54.8%)	31/56 (55.4%)	NS
Weight for age[1] > -1 SD	n (%)	371/481 (77.1%)	336/438 (76.7%)	35/56 (62.5%)	0.032
Weight for age -2 to -1 SD	n (%)	68/481 (14.1%)	55/438 (12.6%)	13/56 (23.2%)	0.038

[1] Weight was not recorded in 11 children and 3 children were oedematous.

For peer review only - http://bmjopen.bmj.com/site/about/guidelines.xhtml

For peer review only

Weight for age -3 to -2 SD	n (%)	34/481 (7.1%)	27/438 (6.1%)	7/56 (12.5%)	NS
Weight for age < -3 SD	n (%)	8/481 (1.7%)	8/438 (1.8%)	0/56 (0%)	NS
Respiratory rate (bpm)	Median [IQR]	60 [54-65]	59 [52.5-64]	68 [62-72]	<0.0001
Body temperature (°C)	Median [IQR]	37.4 [36.8-38.2]	37.4 [36.8-38.1]	37.8 [37.1-38.6]	0.015
Antibiotics given	n (%)	442/466 (94.8%)	390/411 (94.9%)	52/55 (94.6%)	NS
Antimalarial given	n (%)	41/441 (9.3%)	34/392 (8.7%)	7/49 (14.3%)	NS
HIV positive[3]	n (%)	11/494 (2.2%)	10/438 (2.2%)	1/56 (1.8%)	NS
Malaria RDT positive	n (%)	70/485 (14.3%)	59/438 (13.5%)	11/56 (19.6%)	NS
**Clinical Characteristics**					
WHO defined severity[2]	n (%)	64/494 (12.9%)	47/438 (10.7%)	17/56 (30.4%)	<0.001
Fever	n (%)	470/494 (95.1%)	417/438 (95.2%)	53/56 (94.6%)	NS
Cough	n (%)	492/494 (99.6%)	436/438 (99.5%)	56/56 (100%)	NS
Vomiting	n (%)	122/494 (24.7%)	99/438 (22.6%)	23/56 (41.1%)	0.005

[2] Describes 64 children with any one of severe malnutrition (weight for age < 3SD, n=8), unable to feed/drink (n=50), stridor (n=9), lethargy (n=0), unconsciousness (n=0), vomiting everything (n=0), convulsions (n=0).
[3] 184 consented to testing

For peer review only - http://bmjopen.bmj.com/site/about/guidelines.xhtml

Diarrhoea	n (%)	22/494 (4.5%)	19/438 (4.3%)	3/56 (5.4%)	NS
Abnormal sleepiness	n (%)	8/494 (1.7%)	6/438 (1.4%)	2/56 (3.6%)	NS
Coryza	n (%)	259/494 (52.4%)	230/438 (52.5%)	29/56 (51.9%)	NS
Sneezing	n (%)	156/494 (31.6%)	144/438 (32.9%)	12/56 (21.4%)	NS
Difficulty breathing	n (%)	184/494 (37.2%)	138/438 (31.5%)	46/56 (82.1%)	<0.0001
Deep breathing	n (%)	53/494 (34.8%)	26/438 (5.9%)	27/56 (48.2%)	<0.0001
Wheeze	n (%)	36/494 (7.3%)	25/438 (5.7%)	11/56 (19.6%)	0.001
In-drawing lower chest wall	n (%)	172/494 (34.8%)	128/438 (29.2%)	44/56 (78.6%)	<0.0001
Nasal flaring	n (%)	216/494 (43.7%)	175/438 (40.0%)	41/56 (73.2%)	<0.0001
Grunting	n (%)	28/494 (5.6%)	14/438 (3.2%)	14/56 (25.0%)	<0.0001
Head nodding	n (%)	17/494 (3.4%)	10/438 (2.2%)	7/56 (12.5%)	0.001

Abbreviations: bpm, breaths per minute; C, Celsius; HIV human immunodeficiency virus; RDT, rapid diagnostic test.

For peer review only

For peer review only - http://bmjopen.bmj.com/site/about/guidelines.xhtml

Table 2: Microbiologic analyses

		Total Population	Not hospitalised	Hospitalised	
**Bacterial[3]**		**N=488**	**(n=432)**	**(n=56)**	
S. pneumoniae	n (%)	4/494 (0.8%)	4/438 (0.9%)	0/56 (0 %)	NS
S. aureus	n (%)	2/494 (0.4%)	2/438 (0.5%)	0/56 (0%)	NS
Haemophilus spp.	n (%)	2/494 (0.4%)	2/438 (0.5%)	0/56 (0%)	NS
M. pneumoniae	n (%)	3/494 (0.6%)	3/438 (0.7%)	0/56 (0%)	NS
C._pneumoniae	n (%)	2/494 (0.4%)	2/438 (0.5%)	0/56 (0%)	NS
Any bacterial infection	n (%)	13/494 (2.6%)	13/438 (3.0%)	0/56 (0%)	NS
**Viral[4]**					
Adenovirus	n (%)	82/494 (16.6%)	71/438 (16.2%)	11/56 (19.6%)	NS
Bocavirus	n (%)	100/494 (20.2%)	92/438 (21%)	8/56 (20.5%)	NS

[3] Two other bacterial species were identified in the patients who were not hospitalized, but were not clinically associated with pneumonia (E. Coli, and Shigella).
[4] In 6 cases it was not possible to obtain a valid PCR run

For peer review only - http://bmjopen.bmj.com/site/about/guidelines.xhtml

CoV* HKU	n (%)	0/494 (0%)	0/438 (0%)	0/56 (0%)	NS
CoV* NL63	n (%)	1/494 (0.2%)	1/438 (0.2%)	0/56 (0%)	NS
CoV* 229E	n (%)	1/494 (0.2%)	1/438 (0.2%)	0/56 (0%)	NS
CoV* OC43	n (%)	1/494 (0.2%)	1/438 (0.2%)	0/56 (0%)	NS
Influenza A	n (%)	76/494 (15.4%)	74/438 (16.9%)	2/56 (15.6%)	NS
Influenza AH1	n (%)	64/494 (13%)	63/438(14.4%)	1/56 (1.8%)	0.00 1
Influenza AH3	n (%)	6/494 (1.2%)	6/438 (1.4%)	0/56 (0%)	NS
Influenza B	n (%)	9/494 (1.8%)	8/438 (1.8%)	1/56 (1.8%)	NS
HMPV+	n (%)	73/494 (14.7%)	63/438 (14.4%)	10/56 (15.0%)	NS
Parainfluenza 1	n (%)	11/494 (2.2%)	10/438 (2.3%)	1/56 (1.8%)	NS
Parainfluenza 2	n (%)	10/494 (2.0%)	10/438 (2.3%)	0/56 (0%)	NS
Parainfluenza 3	n (%)	4/494 (0.8%)	3/438 (0.7%)	1/56 (1.8%)	NS
Parainfluenza 4	n (%)	17/494 (3.4%)	15/438 (3.4%)	2/56 (3.6%)	NS
RSV// A	n (%)	164/494 (33.2%)	144/438 (32.9%)	20/56 (35.7%)	NS
RSV// B	n (%)	50/488 (10.1%)	44/438(10%)	6/56 (10.7%)	NS
Rhino/enterovirus	n (%)	295/494 (59.7%)	263/438 (60%)	32/56 (57.1%)	NS
Any virus	n (%)	940/488448/ 494 (93.1%)	409/432 (94.7%)	51/56 (91.1%)	NS

* = Coronavirus

+ = Human metapneumovirus

// = Respiratory syncytial virus

For peer review only

For peer review only

For peer review only - http://bmjopen.bmj.com/site/about/guidelines.xhtml

Table 3: Sensitivity and Specificity of WHO Markers of Severe Pneumonia for hospitalization

**Variable**	**Sensitivity (CI 95%)**	**Specificity (CI 95%)**
Severe pneumonia	0.304 (0.188, 0.441)	0.9 (0.868, 0.926)
Vomiting everything	-	0.996 (0.984, 0.999)
Convulsions	-	1 (0.992, 1.000)
Lethargy	-	1 (0.992, 1.000)
Unconsciousness	-	1 (0.992, 1.000)
Malnourished	-	0.987 (0.971, 0.995)
Unable to feed	0.214 (0.116, 0.344)	0.916 (0.886, 0.940)
Stridor	0.089 (0.030, 0.196)	0.991 (0.977, 0.998)
Any sign	0.179 (0.089, 0.304)	0.938 (0.911, 0.958)

For peer review

For peer review only - http://bmjopen.bmj.com/site/about/guidelines.xhtml

Contributorship Statement:

Contributors JG and CW conceived the paper, assisted with study design and secured funding. ML performed data analysis, interpretation and drafted the manuscript. SD, RD, NG, CDG, MC, DW and JW assisted with study design, analysis and interpretation of data. All authors contributed to manuscript development, edited for critical content, and have approved the final version.

Competing Interest Statements:

The authors declare that there are no competing interests of any nature, financial or otherwise.

Funding:

This work was conducted with the generous support of the Bill & Melinda Gates Foundation – Investment ID: OPP1139557

Data Sharing Statement:

Study data pertaining to the BIOTOPE Cohort, may be made available upon reasonable request from the corresponding author.

For peer review only - http://bmjopen.bmj.com/site/about/guidelines.xhtml

Ethics Statement:

The protocol and related documents were approved by the National Health Science Research Committee of Malawi and the Mzuzu Central Hospital Research Committee. Patients received timely treatment and supportive care as required. The study was conducted in compliance with the ethical standards as outlined by two ethics committees and was aligned with the values outlined by the Declaration of Helsinki. (National Health Research Committee of Malawi Ethics Approval #15/11/1532).

References:

1. Liu L, Oza S, Hogan D, Perin J, Rudan I, Lawn JE, et al. Global, regional, and national causes of child mortality in 2000-13, with projections to inform post-2015 priorities: an updated systematic analysis. Lancet. 2015;385(9966):430-40.

2. Manenzhe RI, Zar HJ, Nicol MP, Kaba M. The spread of carbapenemase-producing bacteria in Africa: a systematic review. J Antimicrob Chemother. 2015;70(1):23-40.

3. Carattoli A. Plasmids and the spread of resistance. Int J Med Microbiol. 2013;303(6-7):298-304.

4. Baker KS, Dallman TJ, Ashton PM, Day M, Hughes G, Crook PD, et al. Intercontinental dissemination of azithromycin-resistant shigellosis through sexual transmission: a cross-sectional study. The Lancet Infectious diseases. 2015;15(8):913-21.

For peer review only

5. Revised WHO Classification and Treatment of Pneumonia in Children at Health Facilities: Evidence Summaries. WHO Guidelines Approved by the Guidelines Review Committee. Geneva2014.

6. Hammitt LL, Kazungu S, Morpeth SC, Gibson DG, Mvera B, Brent AJ, et al. A preliminary study of pneumonia etiology among hospitalized children in Kenya. Clin Infect Dis. 2012;54 Suppl 2:S190-9.

7. McCollum ED, King C, Deula R, Zadutsa B, Mankhambo L, Nambiar B, et al. Pulse oximetry for children with pneumonia treated as outpatients in rural Malawi. Bull World Health Organ. 2016;94(12):893-902.

8. Organisation WH. WHO and UNICEF estimates of national immunization coverage 2020 [Available from: https://www.who.int/immunization/monitoring_surveillance/data/mwi.pdf.

9. D'Acremont V, Kilowoko M, Kyungu E, Philipina S, Sangu W, Kahama-Maro J, et al. Beyond malaria--causes of fever in outpatient Tanzanian children. N Engl J Med. 2014;370(9):809-17.

10. Harris PA, Taylor R, Thielke R, Payne J, Gonzalez N, Conde JG. Research electronic data capture (REDCap)--a metadata-driven methodology and workflow process for providing translational research informatics support. Journal of biomedical informatics. 2009;42(2):377-81.

11. Keogh C, Wallace E, O'Brien KK, Galvin R, Smith SM, Lewis C, et al. Developing an international register of clinical prediction rules for use in primary care: a descriptive analysis. Ann Fam Med. 2014;12(4):359-66.

12. Abers MS, Musher DM. Clinical prediction rules in community-acquired pneumonia: lies, damn lies and statistics. QJM. 2014;107(7):595-6.

For peer review only

For peer review only - http://bmjopen.bmj.com/site/about/guidelines.xhtml

13. Fitzwater SP, Chandran A, Santosham M, Johnson HL. The worldwide impact of the seven-valent pneumococcal conjugate vaccine. Pediatr Infect Dis J. 2012;31(5):501-8.

14. Theodoratou E, Johnson S, Jhass A, Madhi SA, Clark A, Boschi-Pinto C, et al. The effect of Haemophilus influenzae type b and pneumococcal conjugate vaccines on childhood pneumonia incidence, severe morbidity and mortality. Int J Epidemiol. 2010;39 Suppl 1:i172-85.

15. Ginsburg AS, Mvalo T, Nkwopara E, McCollum ED, Ndamala CB, Schmicker R, et al. Placebo vs Amoxicillin for Nonsevere Fast-Breathing Pneumonia in Malawian Children Aged 2 to 59 Months: A Double-blind, Randomized Clinical Noninferiority Trial. JAMA Pediatr. 2018.

16. Pneumonia Etiology Research for Child Health Study G. Causes of severe pneumonia requiring hospital admission in children without HIV infection from Africa and Asia: the PERCH multi-country case-control study. Lancet. 2019.

17. Gilani Z, Kwong YD, Levine OS, Deloria-Knoll M, Scott JA, O'Brien KL, et al. A literature review and survey of childhood pneumonia etiology studies: 2000-2010. Clin Infect Dis. 2012;54 Suppl 2:S102-8.

18. Agweyu A, Lilford RJ, English M, Clinical Information Network Author G. Appropriateness of clinical severity classification of new WHO childhood pneumonia guidance: a multi-hospital, retrospective, cohort study. The Lancet Global health. 2018;6(1):e74-e83.

19. Harris M, Clark J, Coote N, Fletcher P, Harnden A, McKean M, et al. British Thoracic Society guidelines for the management of community acquired pneumonia in children: update 2011. Thorax. 2011;66 Suppl 2:ii1-23.

For peer review only

For peer review only - http://bmjopen.bmj.com/site/about/guidelines.xhtml

20. Jain S, Self WH, Wunderink RG, Fakhran S, Balk R, Bramley AM, et al. Community-Acquired Pneumonia Requiring Hospitalization among U.S. Adults. N Engl J Med. 2015;373(5):415-27.

21. Costa LF, Queiroz DA, Lopes da Silveira H, Bernardino Neto M, de Paula NT, Oliveira TF, et al. Human rhinovirus and disease severity in children. Pediatrics. 2014;133(2):e312-21.

Figure Legends

Figure 1: Participant Flow Diagram

For peer review only

For peer review only - http://bmjopen.bmj.com/site/about/guidelines.xhtml

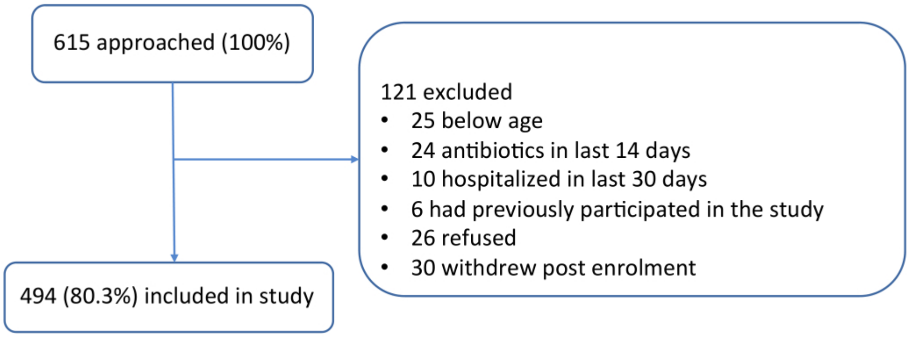

Figure 1: Participant Flow Diagram

402x165mm (72 x 72 DPI)

Supplemental file

Aetiology and Severity of Childhood Pneumonia in Primary Care in Malawi: A Cohort Study

For peer review only

Table of contents

S1: Study Location and Background health data **3**

S2: Microbiology **4**
S2a Microbiology techniques 4
Table S1 Characteristics of individual children with bacterial pneumonia 5
Table S2 Microbiology Results By month 6
Table S3 Microbiology Results By age 7

S3: Markers of severity **8**
Figure S1 Difficulty DRAWING score 8

S4: List of BIOTOPE team & Primary Affiliation **8**

For peer review only

S1: Study Location and Background health data

Mzuzu is one of the four cities in Malawi, with a population of 221,272 (National Statistics Office, 2018). Mzuzu health centre also known as Mapale or Mzimba North Health centre is stituated at the City centre of Mzuzu city while Mzuzu Central Hospital is situated at around 4 km Northwards of the city. The health centre currently serves almost the whole city as a primary health care point while the central hospital is regional referral tertiary hospital serving six districts in the northern region, over hundred health centres and serving a population of over 1.9 million people. It also serves as a secondary healthcare facility for the local (Mzuzu city) population. The majority (98.0%) of our participants were from Mapale health centre. The Central hospital initially served as a primary healthcare facility also but prior to commencement of the study the local health authority decided to maintain it as a secondary care facility only. Therefore it introduced a "bypass fee" where patients who attended the hospital clinics without being referred from a health centre/district hospital were charged MK 1,500 (approxmately 2 Euros). This led to Mapale becoming the main site of recruitment due to the focus on recruiting patients from primary care and before they had received any treatment.

UNICEF data from 2013 reports an under five mortality rate of 68 per 1,000 live births and under one mortality rate of 44 per 1,000 live births in Malawi. In 2016 the estimated coverage was 83% for pneumococcal conjugate vaccine and 84% for Haemophilus influenza type B vaccine in Malawi,[11]

HIV prevalence in antenatal clinics in the Mzimba district (where Mzuzu is located) among those who accepted testing was 9.1%[1]. A study in 2011 in Malawi showed that of 5,068 samples from infants <3 months of age , 764 were ELISA positive indicating 15.1% (14.1–16.1%) of mothers were HIV-infected and passed antibodies to their infant. Sixty-five of the ELISA-positive samples tested positive by DNA PCR, indicating a vertical transmission rate of

8.5% (6.6–10.7%). Survey data indicates 64.8% of HIV-infected mothers and 46.9% of HIV-exposed infants received some form of antiretroviral prophylaxis[2].

S2: Microbiology

S2a Microbiology techniques

Blood cultures

The blood culture samples were inoculated into the Bactec Peds Plus/F Culture vial (Becton Dickinson Diagnostic Instrument Systems). These bottles were incubated in an automated blood culture Bactec™ 9050 which was set at protocol limits of maximum of 7 days to declare the sample negative if no growth has been detected. All positive blood cultures were further subcultured on to agar plates and identified using traditional phenotypic methods (Blood agar, MacConkey and Chocolate agar plate) and Vitek 2 microbial detection system (Biomerieux)

Bacterial PCR

The Masterpure™ complete DNA and RNA purification kit as well as QIA Symphony Automated DNA extraction system [manufacturer. Country] which is an automated method was used to extract DNA and RNA. The extracted DNA was run on AB7500 instruments as per previously detailed methods [4,5]

Viral RT-PCR

Viral RT-PCR was performed on nasopharyngeal and oropharyngeal swabs using a Luminex® MAGPIX® instrument [manufacturer, country] with xPONENT® and SYNCT™ software ,in line with manufacturer's instructions. The panel used was NxTAG respiratory pathogen. The

For peer review

pathogens targeted in this assay were; Influenza A, Influenza A H1, Influenza B, Influenza A H3, Respiratory Syncytial Virus A, Respiratory Syncytial Virus B, Coronavirus 229E, Coronavirus OC43, Coronavirus NL63, Coronavirus HKU1, Human Metapneumovirus, Rhinovirus/Enterovirus Adenovirus, Parainfluenza 1, Parainfluenza 2, Parainfluenza 3, Parainfluenza 4, Human Bocavirus, *Chlamydophila pneumoniae, Mycoplasma pneumoniae, Legionella pneumophila*

Table S1 Characteristics of individual children with bacterial pneumonia

**Bacteria**	**Method of detection**	**Age (years)**	**Gender**	**Malaria**	**HIV**	**Antibiotic**
S. pneumoniae & Haemophilus spp.	Blood PCR	1.1	M	Neg	Neg	Cotrimoxazole
S. pneumoniae	Blood PCR	2.3	M	Pos	Neg	Cotrimoxazole
S. pneumoniae & Haemophilus spp.	Blood PCR	1.1	M	Pos	Neg	Cotrimoxazole
S. pneumoniae & Haemophilus spp.	Blood PCR	1.7	M	Neg	Neg	Unknown
S aureus	Blood culture	2.4	M	Neg	Neg	Cotrimoxazole
S aureus	Blood culture	1.7	M	Neg	Neg	Cotrimoxazole
Haemophilus spp.	Blood PCR	2.8	F	Neg	Neg	Unknown
Haemophilus spp.	Blood PCR	2.7	M	Neg	Neg	Amoxicillin
C. pneumoniae	OP/NP swab PCR	3.6	M	Neg	Neg	Amoxicillin
M. pneumoniae	OP/NP swab PCR	2.6	F	Neg	Pos	Amoxicillin
M. pneumoniae	OP/NP swab PCR	1.7	M	Neg	Neg	Amoxicillin

For peer review only

M. pneumoniae	OP/NP swab PCR	2.4	F	Neg	Neg	Unknown
C. pneumoniae	OP/NP swab PCR	0.6	M	Neg	Unk	Amoxicillin

Table S2 Microbiology Results By month

Variable	Statistic	Mar	Apr	May	Jun
**Bacterial**					
S. pneumoniae	n (%)	0/12 (0%)	0/40 (0%)	3/185 (1.6%)	1/257 (0.4%)
S. auerus	n (%)	0/12 (0%)	0/40 (0%)	0/185 (0%)	2/257 (0.4%)
Haemophilus spp.	n (%)	0/12 (0%)	0/40 (0%)	2/185 (1.1%)	3/257 (1.2%)
M. pneumoniae	n (%)	0/12 (0%)	1/40 (2.5%)	2/185 (1.1%)	0/257 (0%)
C._pneumoniae	n (%)	0/12 (0%)	0/40 (0%)	1/185 (0.5%)	1/257 (0.4%)
Other_culture	n (%)	0/12 (0%)	0/40 (0%)	1/185 (0.5%)	2/257 (0.8%)
**Viral**[1]					
Adenovirus	n (%)	6/12 (50%)	10/40 (25%)	33/185 (17.8%)	30/257 (11.7%)
Bocavirus	n (%)	2/12 (16.7%)	7/40 (17.5%)	37/185 (20%)	47/257 (18.3%)
CoV* HKU	n (%)	0/12 (0%)	0/40 (0%)	0/185 (0%)	0/257 (0%)
CoV* NL63	n (%)	0/12 (0%)	1/40 (2.5%)	0/185 (0%)	0/257 (0%)
CoV* 229E	n (%)	0/12 (0%)	0/40 (0%)	0/185 (0%)	1/257 (0.4%)
CoV* OC43	n (%)	0/12 (0%)	0/40 (0%)	0/185 (0%)	1/257 (0.4%)
Influenza A	n (%)	0/12 (0%)	10/40 (25%)	42/185 (22.7%)	23/257 (8.9%)
Influenza AH1	n (%)	0/12 (0%)	10/40 (25%)	37/185 (20%)	16/253 (6.2%)
Influenza AH3	n (%)	0/12 (0%)	0/40 (0%)	3/185 (1.6%)	3/257 (1.2%)
Influenza B	n (%)	0/12 (0%)	0/40 (0%)	2/185 (1%)	6/257 (2.3%)
HMPV+	n (%)	2/12 (16.7%)	10/40 (25%)	33/185 (17.8%)	27/257 (10.5%)

For peer review only

Parainfluenza 1	n (%)	0/12 (0%)	2/40 (5%)	5/182 (2.7%)	4/257 (1.5%)
Parainfluenza 2	n (%)	0/12 (0%)	3/40 (7.5%)	4/185 (2.2%)	3/257 (1.2%)
Parainfluenza 3	n (%)	1/12 (8.3%)	0/40 (0%)	3/185 (1.6%)	0/257 (0%)
Parainfluenza 4	n (%)	1/12 (8.3%)	3/40 (7.5%)	7/185 (3.8%)	5/257 (1.9%)
RSV// A	n (%)	0/12 (0%)	1/40 (2.5%)	22/185 (11.9%)	129/257 (50.2%)
RSV// B	n (%)	0/12 (0%)	2/40 (5%)	12/185 (6.5%)	35/257 (13.6%)
Rhino/enterovirus	n (%)	10/12 (83.3%)	22/40 (55%)	132/185 (71.3%)	128/257 (49.8%)

* = Coronavirus

+ = Human metapneumovirus

// = Respiratory syncytial virus

Table S3 Microbiology Results By age

Variable	Statistic	2-11 months	12-35 months	36+ months
**Bacterial**				
S. pneumoniae	n (%)	2/157 (1.3%)	2/252 (0.8%)	0/85 (0%)
S. auerus	n (%)	0/157 (0%)	2/252 (0.8%)	0/85 (0%)
Haemophilus spp.	n (%)	2/157 (1.4%)	3/252 (1.2%)	0/85 (0%)
M. pneumoniae	n (%)	0/157 (0%)	3/252 (1.2%)	0/85 (0%)
C. pneumoniae	n (%)	0/157 (0%)	1/252 (0.4%)	1/85 (1.2%)
**Viral**				
Adenovirus	n (%)	12/157 (7.6%)	48/252 (19%)	17/85 (20%)
Bocavirus	n (%)	25/157 (15.9%)	52/252 (20.6%)	14/85 (16.5%)
CoV* HKU	n (%)	0/157 (0%)	0/252 (0%)	0/85 (0%)
CoV* NL63	n (%)	0/157 (0%)	1/252 (0.4%)	0/85 (0%)
CoV* 229E	n (%)	0/157 (0%)	1/252 (0.4%)	0/85 (0%)
CoV* OC43	n (%)	0/157 (0%)	1/252 (0.4%)	0/85 (0%)
Influenza A	n (%)	8/157 (5%)	40/252 (15.9%)	26/85 (30.6%)
Influenza AH1	n (%)	8/157 (5%)	30/252 (11.9%)	24/85 (28.2%)
Influenza AH3	n (%)	0/157 (0%)	5/252 (1.9%)	1/85 (1.2%)
Influenza B	n (%)	2/157 (1.3%)	3/252 (1.2%)	2/84 (2.3%)
HMPV+	n (%)	19/157 (12.1%)	41/252 (16.3%)	11/84 (12.9%)
Parainfluenza 1	n (%)	2/157 (1.3%)	4/252 (1.6%)	5/85 (5.8%)
Parainfluenza 2	n (%)	2/157 (1.3%)	5/252 (2%)	2/85 (2.3%)
Parainfluenza 3	n (%)	1/157 (0.6%)	3/252 (1.2%)	0/85 (0%)

For peer review only

Parainfluenza 4	n (%)	4/157 (2.5%)	9/252 (3.6%)	3/85 (3.5%)
RSV// A	n (%)	61/157 (38.8%)	76/252 (30.2%)	10/85 (11.8%)
RSV// B	n (%)	18/157 (11.4%)	22/252 (8.7%)	7/85 (8.2%)
Rhino/enterovirus	n (%)	83/157 (52.9%)	148/252 (58.7%)	53/85 (62.3%)

* = Coronavirus

+ = Human metapneumovirus

// = Respiratory syncytial virus

S3: Markers of severity

Figure S1 Difficulty DRAWING Breath score and risk of hospitalisation

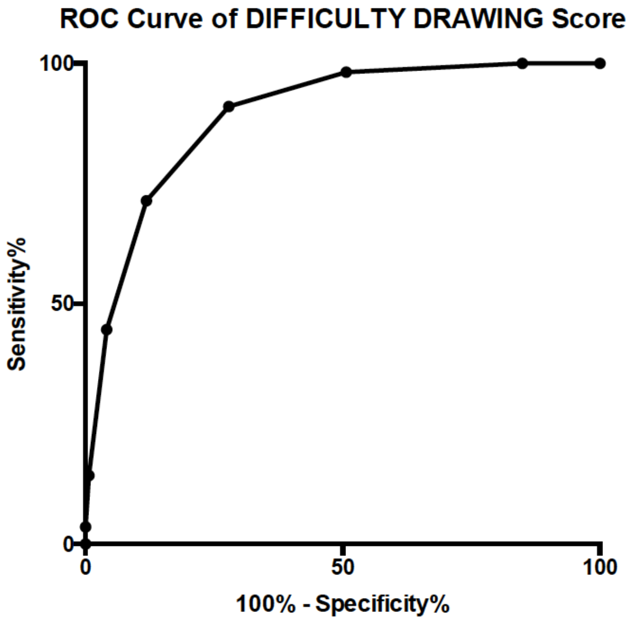

DIFFICULTY DRAWING breath score = 1 for each of:

DIFFICULTY breathing
Deep breathing
Respiratory Rate > 70 bpm

Age <2 years
Wheeze
INdrawing lower chest wall
Grunting

Area under the ROC curve
Area 0.8918
Std. Error 0.02002
95% confidence interval 0.8525 to 0.9310
P value< 0.0001
Data
Controls (Not Hospitalised) 438
Patients (Hospitalised) 56
Missing Controls0
Missing Patients0

[1] The DD Score (strongly, positively associated) and previous number of vaccines (modestly, negatively associated) are the only independent predictors of hospitalisation in the model with the following covariates included:

- Staff designation of severe pneumonia (IMCI criteria)
- Age
- HIV status (immunosuppression)
- Completed WHO vaccination schedule
- Number sleeping in same room as child (overcrowding)
- Chimney present for indoor fire (indoor smoke)
- Electricity present (marker of socioeconomic status)
- Malaria co –infection (disease severity)
- Distance to nearest clinic (marker of healthcare access)

For each unit increase in the DD Score there is a 10.6% (95% CI 7.9-13.4) increase in the likelihood of severe disease as defined by hospitalisation (p<0.0001)

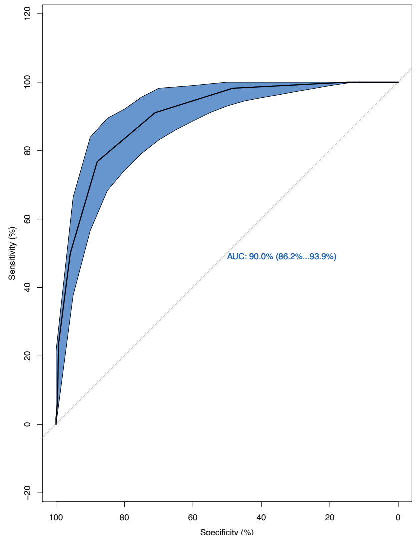

[2] The DD Score is the only strong, independent predictor of severe pneumonia as designated by the staff in a similar model with the following covariates included:

- Hospitalisation
- Age
- HIV status (immunosuppression)
- Completed WHO vaccination schedule
- Number sleeping in same room as child (overcrowding)
- Chimney present for indoor fire (indoor smoke)
- Electricity present (marker of socioeconomic status)
- Malaria co –infection (disease severity)
- Distance to nearest clinic (marker of healthcare access)

For each unit increase in the DD Score there is a 6.0% (95% CI 2.5-9.6) increase in the likelihood of severe disease as defined by WHO criteria ($p<0.001$)

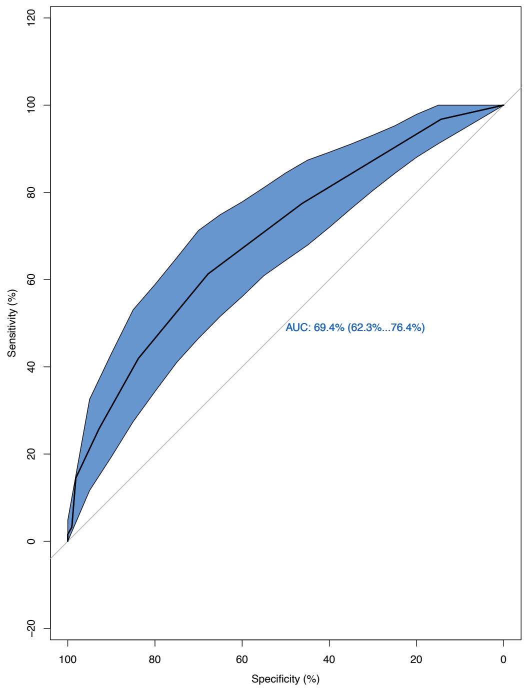

Area under the ROC curve
Area 0.69
95% confidence interval 0.62 to 0.76
P value< 0.001

S4: List of BIOTOPE team & Primary Affiliation

gHealth Research Group, School of Medicine, UCD
Joe Gallagher
Nadezhda Glezeva
Mark Ledwidge (statistics)

John O Donoghue
Dermot Wildes

Irish Meningitis and Sepsis Reference Laboratory
Richard Drew
Desiree Bennett
Nicola O'Sullivan

Luke International
Joseph Wu
Hsin yi Lee

Mzuzu Central Hospital/Mapale Health Centre
Master Chisale
Maynard Khondowe,
Themba Luwe
Hastings Scott
Chikondi Chimbatata
Cecilia Mwalilino
Kelvin Ulili
Lameck Manda
Joseph Phiri
Alinafe Mpicha
Isaac Namwiri

National Virus Reference Laboratory, Ireland
Cillian De Gascun
Suzie Coughlan
Linda Dunford
Christina Byrne

Queens University Belfast
Chris Watson

Royal College of Surgeons in Ireland
Sudip Das

Eoin O Connell (statistics)

For peer review only

1. Manda S, Masenyetse L, Cai B, Meyer R. Mapping HIV prevalence using population and antenatal sentinel-based HIV surveys: a multi-stage approach. Popul Health Metr 2015;13:22.
2. Sinunu MA, Schouten EJ, Wadonda-Kabondo N, et al. Evaluating the impact of prevention of mother-to-child transmission of HIV in Malawi through immunization clinic-based surveillance. PloS one 2014;9:e100741.
3. International. NSONMaI. Malawi Demographic and Health Survey 2015-16: Key Indicators Report. 2016.
4. Meyler KL, Meehan M, Bennett D, Cunney R, Cafferkey M. Development of a diagnostic real-time polymerase chain reaction assay for the detection of invasive Haemophilus influenzae in clinical samples. Diagn Microbiol Infect Dis. 2012 Dec;74(4):356-62. doi: 10.1016/j.diagmicrobio.2012.08.018. Epub 2012 Sep 25. PMID: 23017260.
5. [1]

Murphy J, O' Rourke S, Corcoran M, O' Sullivan N, Cunney R, Drew R. Evaluation of the Clinical Utility of a Real-time PCR Assay for the Diagnosis of Streptococcus pneumoniae Bacteremia in Children: A Retrospective Diagnostic Accuracy Study. Pediatr Infect Dis J. 2018 Feb;37(2):153-156. doi: 10.1097/INF.0000000000001772. PMID: 29076932.

review only

STROBE Statement—checklist of items that should be included in reports of observational studies

	Item No.	Recommendation	Page No.
**Title and abstract**	1	(*a*) Indicate the study's design with a commonly used term in the title or the abstract	1
		(*b*) Provide in the abstract an informative and balanced summary of what was done and what was found	3
**Introduction**			
Background/rationale	2	Explain the scientific background and rationale for the investigation being reported	5
	3	State specific objectives, including any prespecified hypotheses	6
**Methods**			
Study design	4	Present key elements of study design early in the paper	6
Setting	5	Describe the setting, locations, and relevant dates, including periods of recruitment, exposure, follow-up, and data collection	6
Participants	6	(*a*) *Cohort study*—Give the eligibility criteria, and the sources and methods of selection of participants. Describe methods of follow-up	6-7
Variables	7	Clearly define all outcomes, exposures, predictors, potential confounders, and effect modifiers. Give diagnostic criteria, if applicable	8
Data sources/ measurement	8*	For each variable of interest, give sources of data and details of methods of assessment (measurement). Describe comparability of assessment methods if there is more than one group	*8*
Bias	9	Describe any efforts to address potential sources of bias	
Study size	10	Explain how the study size was arrived at	9

Continued on next page

For peer review only

Quantitative variables	11	Explain how quantitative variables were handled in the analyses. If applicable, describe which groupings were chosen and why	
Statistical methods	12	(*a*) Describe all statistical methods, including those used to control for confounding	9
		(*b*) Describe any methods used to examine subgroups and interactions	
		(*c*) Explain how missing data were addressed	9
		(*d*) *Cohort study*—If applicable, explain how loss to follow-up was addressed	9
		(*e*) Describe any sensitivity analyses	NA
**Results**			
Participants	13*	(a) Report numbers of individuals at each stage of study—eg numbers potentially eligible, examined for eligibility, confirmed eligible, included in the study, completing follow-up, and analysed	Figure 1
		(b) Give reasons for non-participation at each stage	Figure 1
		(c) Consider use of a flow diagram	Figure 1
Descriptive data	14*	(a) Give characteristics of study participants (eg demographic, clinical, social) and information on exposures and potential confounders	Table 1
		(b) Indicate number of participants with missing data for each variable of interest	Table 1
		Summarise follow-up time (eg, average and total amount)	8
Outcome data	15*	Report numbers of outcome events or summary measures over time	*Table 1*
Main results	16	(*a*) Give unadjusted estimates and, if applicable, confounder-adjusted estimates and their precision (eg, 95% confidence interval). Make clear which confounders were adjusted for and why they were included	10-12
		(*b*) Report category boundaries when continuous variables were categorized	
		(*c*) If relevant, consider translating estimates of relative risk into absolute risk for a meaningful time period	Not applicable

Continued on next page

For peer review only - http://bmjopen.bmj.com/site/about/guidelines.xhtml

For peer review only

Other analyses	17	Report other analyses done—eg analyses of subgroups and interactions, and sensitivity analyses	
**Discussion**			
Key results	18	Summarise key results with reference to study objectives	12
Limitations	19	Discuss limitations of the study, taking into account sources of potential bias or imprecision. Discuss both direction and magnitude of any potential bias	14
Interpretation	20	Give a cautious overall interpretation of results considering objectives, limitations, multiplicity of analyses, results from similar studies, and other relevant evidence	14-15
Generalisability	21	Discuss the generalisability (external validity) of the study results	14
**Other information**			
Funding	22	Give the source of funding and the role of the funders for the present study and, if applicable, for the original study on which the present article is based	23

*Give information separately for cases and controls in case-control studies and, if applicable, for exposed and unexposed groups in cohort and cross-sectional studies.

Note: An Explanation and Elaboration article discusses each checklist item and gives methodological background and published examples of transparent reporting. The STROBE checklist is best used in conjunction with this article (freely available on the Web sites of PLoS Medicine at http://www.plosmedicine.org/, Annals of Internal Medicine at http://www.annals.org/, and Epidemiology at http://www.epidem.com/). Information on the STROBE Initiative is available at www.strobe-statement.org.

For peer review only
